# Post-CNS-inflammation expression of CXCL12 promotes the endogenous myelin/neuronal repair capacity following spontaneous recovery from multiple sclerosis-like disease

**DOI:** 10.1186/s12974-015-0468-4

**Published:** 2016-01-08

**Authors:** Rina Zilkha-Falb, Nathali Kaushansky, Naoto Kawakami, Avraham Ben-Nun

**Affiliations:** Department of Immunology, The Weizmann Institute of Science, 234 Herzl Street, Rehovot, 7610001 Israel; Institute of Clinical Neuroimmunology, Ludwig-Maximilians-University, 81377 Munich, Germany; Present address: Multiple Sclerosis Center, Neurogenomics Laboratory, Sheba Medical Center, Tel-Hashomer, Israel

**Keywords:** Multiple sclerosis, Experimental autoimmune encephalomyelitis (EAE), CXCL12, Neuronal progenitor cells, Oligodendrocyte precursor cells, Myelin and neuronal repair

## Abstract

**Background:**

Demyelination and axonal degeneration, hallmarks of multiple sclerosis (MS), are associated with the central nervous system (CNS) inflammation facilitated by C-X-C motif chemokine 12 (CXCL12) chemokine. Both in MS and in experimental autoimmune encephalomyelitis (EAE), the deleterious CNS inflammation has been associated with upregulation of CXCL12 expression in the CNS. We investigated the expression dynamics of CXCL12 in the CNS with progression of clinical EAE and following spontaneous recovery, with a focus on CXCL12 expression in the hippocampal neurogenic dentate gyrus (DG) and in the corpus callosum (CC) of spontaneously recovered mice, and its potential role in promoting the endogenous myelin/neuronal repair capacity.

**Methods:**

CNS tissue sections from mice with different clinical EAE phases or following spontaneous recovery and in vitro differentiated adult neural stem cell cultures were analyzed by immunofluorescent staining and confocal imaging for detecting and enumerating neuronal progenitor cells (NPCs) and oligodendrocyte precursor cells (OPCs) and for expression of CXCL12.

**Results:**

Our expression dynamics analysis of CXCL12 in the CNS with EAE progression revealed elevated CXCL12 expression in the DG and CC, which persistently increases following spontaneous recovery even though CNS inflammation has subsided. Correspondingly, the numbers of NPCs and OPCs in the DG and CC, respectively, of EAE-recovered mice increased compared to that of naïve mice (NPCs, *p* < 0.0001; OPCs, *p* < 0.00001) or mice with active disease (OPCs, *p* < 0.0005). Notably, about 30 % of the NPCs and unexpectedly also OPCs (~50 %) express CXCL12, and their numbers in DG and CC, respectively, are higher in EAE-recovered mice compared with naïve mice and also compared with mice with ongoing clinical EAE (CXCL12^+^ NPCs, *p* < 0.005; CXCL12^+^ OPCs, *p* < 0.0005). Moreover, a significant proportion (>20 %) of the CXCL12^+^ NPCs and OPCs co-express the CXCL12 receptor, CXCR4, and their numbers significantly increase with recovery from EAE not only relative to naïve mice (*p* < 0.0002) but also to mice with ongoing EAE (*p* < 0.004).

**Conclusions:**

These data link CXCL12 expression in the DG and CC of EAE-recovering mice to the promotion of neuro/oligodendrogenesis generating CXCR4^+^ CXCL12^+^ neuronal and oligodendrocyte progenitor cells endowed with intrinsic neuro/oligondendroglial differentiation potential. These findings highlight the post-CNS-inflammation role of CXCL12 in augmenting the endogenous myelin/neuronal repair capacity in MS-like disease, likely via CXCL12/CXCR4 autocrine signaling.

**Electronic supplementary material:**

The online version of this article (doi:10.1186/s12974-015-0468-4) contains supplementary material, which is available to authorized users.

## Background

Demyelination and axonal degeneration, the hallmarks of multiple sclerosis (MS), have long been associated with the central nervous system (CNS) inflammation [1]. Chemokines are important mediators of the inflammatory response, and the chemokine-guided influx of inflammatory cells into the CNS is a major contributor to myelin damage and axonal degeneration. The inflammation observed in the CNS of MS patients and of mice with experimental autoimmune encephalomyelitis (EAE) is associated with increased expression of the C-X-C motif chemokine 12 (CXCL12) [2–5]. As a strong chemoattractant for T- and B-lymphocytes and inflammatory monocytes, CXCL12 is regarded as a mediator of pathogenic inflammation in the CNS. Recently, however, the function of CXCL12 in the CNS of mice with EAE, and apparently also of MS patients, was shown to be more complex; depending on its interaction with receptors CXCR4 or CXCR7 and on its spatial and temporal expression, CXCL12 may be anti-inflammatory [4], immunomodulatory [6], and may also promote remyelination [7–10].

CXCL12 is a key pleiotropic chemokine. In addition to its essential function in regulating hematopoiesis and in the patterning of the immune system, it plays an important role in the plasticity and patterning of the CNS [7, 11, 12]. During embryonic development, CXCL12 is crucial for neural stem cells (NSCs) proliferation, survival and migration, intercellular communication, and neuronal axon guidance [7, 11, 13, 14]. CXCL12 and its receptors, CXCR4 or CXCR7, are also expressed in the adult brain [11, 15], where the CXCL12/CXCR4 axis plays a central role in regulating the survival, proliferation, maturation, and migration of NSCs in response to CNS insults, including neuroinflammation-associated disorders, suggesting that CXCL12/CXCR4 axis is important also in adult neuro/oligodendrogenesis following CNS damage [13, 16–20].

Although expression patterns of CXCR4 and its CXCL12 ligand have been extensively investigated during CNS development and in healthy and injured adult CNS [7, 10, 11, 21], few studies have analyzed functions of CXCL12 in the context of CNS demyelinating diseases such as EAE/MS [7, 8, 22]. In relapsing/remitting MS, the most common form of the disease, relapse episodes are associated with demyelination and neuronal damage. Albeit limited, spontaneous remyelination may occur in MS [23] and, more profoundly, in viral model of MS [24]. Remyelination occurs via oligodendrocyte precursor cells (OPCs), which are derived primarily from NSCs that persist in the adult brain in neurogenic niches lining the subventricular zone of the lateral ventricles and the subgranular layer of the hippocampal dentate gyrus (DG). The NSCs are self-renewing and can differentiate to neuronal progenitor cells (NPCs) and to OPCs [7, 11]. The OPCs migrate to demyelinated areas in the CNS where they differentiate into mature myelin-producing oligodendrocytes [7, 25].

It has been proposed that CXCL12 via its CXCR4 receptor plays a role also in regulating the survival and migration of NSCs in response to CNS trauma, such as brain tumors, ischemia, and neuroinflammation-associated disorders [13, 16–20], and in regulating the migration and survival of OPCs in vitro [26, 27]. In vivo, the role of CXCL12 and its CXCR4 receptor in promoting the differentiation of OPCs and their remyelination has been demonstrated in mice following cuprizone-induced demyelination [8] and ischemia-induced demyelination [10]. Yet, the potential effect of CXCL12 on NPCs or OPCs during the clinical course of EAE, a model for MS, and its possible relevance to spontaneous recovery from the disease is yet to be elucidated.

In this study, analysis of the expression dynamics of CXCL12 in the CNS of mice during the progression of clinical EAE and following spontaneous recovery showed that CXCL12 expression, which increased with disease progression, did not decline to basal levels in the CNS of spontaneously recovered mice. In recovered mice, CXCL12 levels remained significantly elevated relative to those in naïve CNS, although CNS inflammation and CXCL12-producing inflammatory cells had been abolished during recovery. The post-inflammatory CXCL12 expression in the CNS of spontaneously recovered mice was associated with a significant increase in numbers of NPCs and OPCs at the DG and corpus callosum (CC), respectively, that expressed the CXCL12. Notably, a significant proportion of the NPCs and OPCs co-expressed CXCL12 and its receptor, CXCR4. The significant increase in numbers of CXCL12^+^ CXCR4^+^ NPCs and OPCs, which have an intrinsic potential to proceed towards differentiation to mature neuronal cells or oligodendrocytes, likely via autocrine signaling mechanisms, links the post-inflammatory expression of CXCL12 with post-CNS-inflammation elevation of endogenous myelin/neuronal repair capacity associated with spontaneous recovery from clinical EAE.

## Methods

### Reagents and antibodies

The mouse PLP139-151 peptide was synthesized in the laboratory of Prof. M. Fridkin (Department of Organic Chemistry, Weizmann Institute of Science), using the Fmoc technique with an automated peptide synthesizer (AMS422; ABIMED, Langenfeld, Germany).

Antibodies used for immunostaining included the following: mouse anti- CXCL12 (clone K15C kindly provided by Prof. Tzvi Lapidot; Weizmann Institute, Israel); rabbit anti-CD3 (NeoMarkers, Fremont, CA); rat anti-mouse MAC2 (Biolegend); monoclonal mouse anti-GFAP (Sigma); rabbit anti-GFAP (Dako); rat anti-MBP (Chemicon); rabbit anti-NF200 (Sigma); goat anti-DCX (Chemicon); monoclonal mouse anti-β-Tubulin III (Chemicon); rabbit anti NG2 (Chemicon); and rat anti-CXCR4 (eBioscience).

The chemokine receptor antagonist, bicyclam AMD3100, was purchased from Sigma-Aldrich (St. Louis, MO, USA).

### Animals

C57Bl/6J and SJL/J mice were purchased from Harlan (Jerusalem, Israel), and (C57Bl/6J × SJL/J) F1 mice were bred and maintained at the Weizmann Institute Animal Facility under specific pathogen-free conditions. All animal procedures and experimental protocols were approved by the IACUC of the Weizmann Institute (permit number 02820711-3) and were performed in compliance with its relevant guidelines and regulations.

### Induction of EAE and tissue processing

For our experiments, PLP/EAE in (C57BL × SJL/J) F1 mice was found to be more suitable model than PLP/EAE in SJL/J mice. In our animal facilities, the disease in SJL/J mice was more severe (and with morbidity) than the disease in the F1 mice. PLP/EAE in (C57BL × SJL/J) F1 mice was with less morbidity and with more mice that recover from the disease spontaneously, mice that were necessary for the experiments in this study. (C57BL × SJL/J) F1 mice (females, 7–8 weeks old) were injected subcutaneously at one site in the flank with 200 μl of emulsion containing PLP139-151 (100 μg) in CFA containing 300 μg mycobacterium tuberculosis H37Ra (Difco). Mice received 300 ng pertussis toxin in 500 μl PBS in the tail vein immediately and 48 h after immunization. Following the encephalitogenic challenge, mice were observed and scored as previously described [28]. For further tissue processing, mice were anesthetized with a xylazine/ketamine mixure and perfused intracardially with cold 4 % paraformaldehyde (PFA). Spinal cords and brains were post-fixed in 2 % PFA and cryoprotected in a 15 % sucrose solution. Free-floating sections (16 μm thick for the spinal cord and 30 μm thick for the brain were cut coronally with a sliding microtome (Leica SM 2000r; Leica, Nussloch, Germany) and stored at 4 °C prior to immunolabeling.

### Immunolabeling and quantification

#### Immunocytochemistry

Coverslips were washed with PBS, fixed with 2 % PFA, treated with a permeabilization/blocking solution [(10 % FCS, 2 % bovine serum albumin, 1 % glycine, and 0.1 % Triton X-100 (Sigma-Aldrich, Rehovot, Israel)] and stained with either of the following antibodies (diluted in the permeabilization/blocking solution): monoclonal anti-mouse GFAP (1:400); rabbit anti-NG2 (1:200); rat anti-MBP (1:300); goat anti-DCX (1:200); monoclonal anti-β-tubulin III (1:500); rabbit anti-NF200 (1:500); and monoclonal anti-CXCL12 (1:84). Cover slips were exposed to primary antibodies for 1 h in a humidified chamber at room temperature. Secondary anti-IgG antibodies used included: Cy3-conjugated goat anti-mouse, Cy3-conjugated goat anti-rabbit, Alexa488-conjugated donkey anti-rabbit, Cy2-conjugated donkey anti-rat, Rhodamine Red™-x-conjugated donkey anti-goat, Cy3-conjugated goat anti-mouse, Cy5-conjugated donkey anti-mouse, and Cy5-conjugated donkey anti-rat. All secondary antibodies were purchased from Jackson ImmunoResearch Laboratories Inc. and used at a dilution of 1:250, except for Rhodamine Red™-x-conjugated donkey anti-goat (1:100). Cover slips were exposed to secondary antibodies for 1 h in a humidified chamber at room temperature. Following the immunostaining, cells were counterstained with 4′,6′-diamidino-3-phenylindole (DAPI) to visualize the nuclei. Control coverslips (not treated with primary antibody) were used to distinguish specific staining from non-specific staining or autofluorescent components.

Slides were examined using an LSM 510 laser scanning confocal microscope (Carl Zeiss, Jena, Germany). Digital images were acquired using the Zeiss LSM 510 software (magnifications ×10, ×25, ×40, or ×63). Images were processed using Photoshop software.

#### Immunofluorescent staining of tissue sections

Free-floating sections of the spinal cord or brain were washed twice in PBS followed by blocking in 3 % rabbit or goat serum and 0.1 % Triton-X-100 in PBS for 1 h at RT. Samples were stained with the following antibodies in 1 % of the appropriate serum and 0.1 % Triton-X-100 in PBS at 4 °C for overnight: monoclonal anti CXCL12 (1:84) with rabbit anti-GFAP (1:200), rabbit polyclonal anti NG2 (1:200), or with goat anti DCX (1:200). In some cases, samples were stained with rat anti MAC2 (1:200), rabbit anti CD3 (1:100), or with rat anti CXCR4 (1:200). For detection of primary antibodies, samples were stained with either of the secondary antibodies described above. Images were acquired as described above.

#### Quantification

Quantification of CXCL12 and the number of MAC2^+^/CD3^+^, GFAP^+^, DCX^+^, and NG2^+^ alone or with CXCL12 at the different stages of EAE was performed on the same groups of animals. For analysis of the brain tissues, CD3^+^ and MAC2^+^ cells were quantified in the forebrain and midbrain and averaged; MAC2^+^ cells were also quantified in the dentate gyrus. Astrocytes were scored in the dentate gyrus by counting GFAP^+^ and GFAP^+^/CXCL12^+^ cells. Neuronal progenitor cells were scored by counting DCX^+^ cells in the subgranular zone (SGZ) of the dentate gyrus as well as DCX^+^/CXCL12^+^ cells. Oligodendrocyte progenitor cells were scored by counting NG2^+^ cells in the corpus callosum as well as NG2^+^/CXCL12^+^ cells. All cell lineages are expressed as mean number of cells per square millimeter. For quantification, 5–6 coronal consecutive sections (at 300 μm intervals) per mouse brain were stained, counted, and calculated for square millimeter. The nuclei of cells were visualized by DAPI counterstaining.

For in vitro analysis, the percentage of antibody-labeled cells was determined by evaluating 800–1000 cells in at least six randomly chosen fields of view (under ×20 objective). All analyses were performed by an observer blinded to identity of the examined slides.

#### Image analysis

Quantitative colocalization analysis has been extensively used to reliably determine the colocalization of proteins. The method used in this study has been previously described [29]. The weighted colocalization coefficient (WCC), which is the sum of intensities of colocalizing pixels relative to the overall sum of pixel intensities above the threshold (or background), was used to determine the relative levels of CXCL12 that colocalized with NG2 immunoreactive cells. This was calculated using the colocalization module according to Manders et al. [30]. The advantage of a weighted colocalization coefficient is that differences in pixel intensity are taken into account (i.e., not all pixels contribute equally to the final colocalization coefficient value).

### Adult neural stem cell culture

The neural stem cells were generated from the subventricular zone (SVZ) of the lateral ventricle from brains of C57Bl/6 mice (5–7 weeks old). Briefly, SVZ was isolated following coronal sectioning and cut into 1 mm^3^ pieces. The tissue was minced and incubated for digestion at 37 °C, 5 % CO_2_ for 45 min in 0.25 % Trypsin-EDTA (Biological industries, Beit-Haemek, Israel). Following centrifugation at 110*g* for 10 min at room temperature, the tissue was further digested in Earle’s balanced salt solution containing 0.94 mg/ml papain (Sigma-Aldrich, Rehovot, Israel) and 0.01 % DNase (Sigma-Aldrich, Rehovot, Israel) for 30 min at 37 °C, 5 % CO_2_. Then, the tissue was mechanically dissociated by pipette trituration. Single-cell suspension were plated (3500 cells/cm^2^) in 75 cm^2^ Falcon tissue culture flasks (BD Biosciences, Franklin Lakes, NJ, USA), in Neurospheres medium [Dulbecco’s modified Eagles’ medium (DMEM):F12 medium (Invitrogen Corp.) supplemented with B27 supplement (Invitrogen Corp.), glucose, Hepes, bFGF (human recombinant, 20 ng/ml) and EGF (mouse recombinant, 20 ng/ml); both from PeproTech, (Rocky hill, NJ, USA)].

Fresh media was added every 3–4 days to maintain the cells as proliferating neurospheres, which were then passaged every 4–6 days and re-plated as single cells.

The neurospheres were differentiated towards different neural lineages by plating cells on Poly-d-lysine [PDL (Sigma-Aldrich, Rehovot, Israel)], in growth factor-free neurosphere medium containing 5 % serum (differentiation medium). For immunocytochemistry, cells were plated on coverslips pre-coated with PDL. In some differentiation experiments, NSCs (2 × 10^4^) were cultured in the presence or absence of 10 ng/ml CXCL12 (PeproTech, Rocky hill, NJ, USA) in differentiation medium. To monitor the effect of the CXCR4 antagonist AMD3100 on differentiation of NSCs, AMD3100 (100 ng/ml, Sigma-Aldrich, Rehovot, Israel) was applied with or without CXCL12 (10 ng/ml) for 4 days in differentiation medium. AMD3100 was replaced twice a day.

### Densitometry and statistical analysis

ImageJ densitometry software (version 1.36, National Institutes of Health, Bethesda, MD, USA) was used for quantification of CXCL12 intensity from images of brain sections. Results are expressed as mean ± SEM. Statistical significance was assessed with an unpaired two-tail Student’s *t* test (Excel software). *P* < 0.05 was considered significant.

## Results

### CXCL12 expression is elevated in the CNS at EAE onset and remains high following spontaneous recovery

**Fig. 1:**
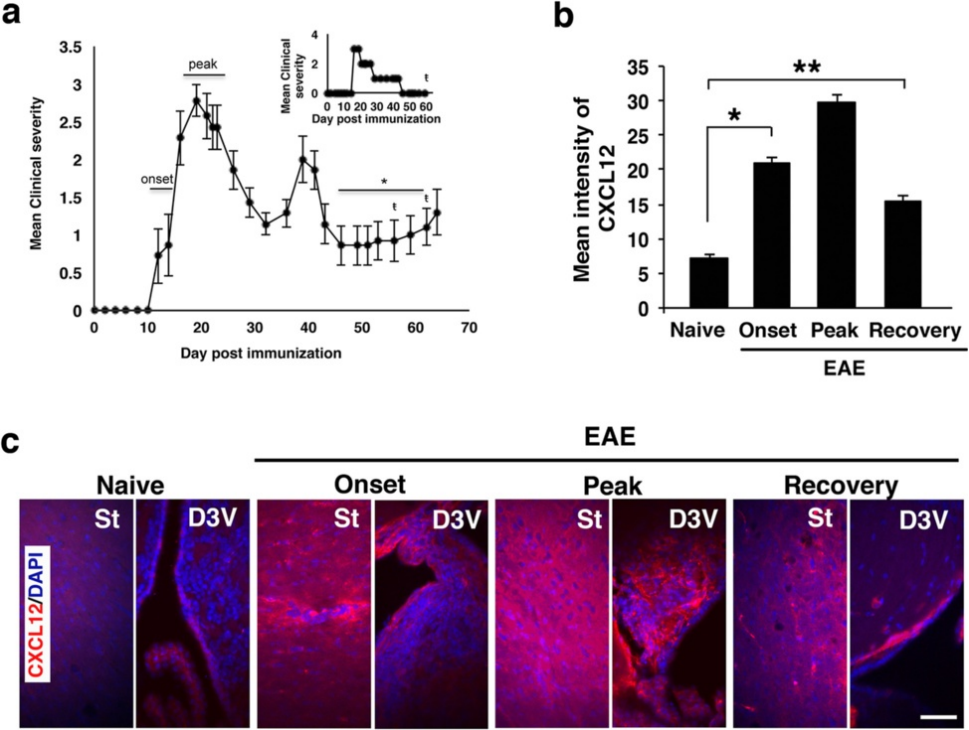
Clinical course of PLP139-151-induced EAE in a representative experiment and sampling CXCL12 expression in the CNS at different phases of EAE. **a** EAE was induced in (C57Bl/6J × SLJ/J) F1 mice (*n* = 20) by immunization with PLP139-151/CFA and scored for the clinical manifestations as described in “Methods” section. The *horizontal bars* indicate phase of disease progression at which mice were sacrificed for immunohistopathology analysis of the CNS. In the second remission phase (denoted by *asterisk*), only individual mice that spontaneously fully recovered from EAE (with a score =0 for 7–10 days) were used for immunohistopathology analysis of the CNS. These mice are referred to as EAE-recovered mice. In this representative experiment, three mice fully recovered from clinical EAE on the days denoted by *ŧ. Inset* represents the clinical follow-up of an individual mouse that spontaneously recovered from clinical EAE on day 48 and sacrificed for immunohistopathology analysis on day 60 post-immunization. **b** Quantification of CXCL12 immunofluorescence intensity in consecutive tissue sections taken from the forebrain, midbrain, and hindbrain from naïve or mice at onset, peak of disease, or following spontaneous recovery (*p < 10^−11^, **p < 10^−9^). **c** Immunostaining for CXCL12 (*red*) in the striatum (*St*) and the dorsal third ventricle (*D3V*). Data in **b** are from nine consecutive sections from each mouse, *n* = 3 mice/group. *Error bars* represent mean ± SEM. All sections were counterstained with DAPI (*blue*) to visualize cell nuclei. Scale bar: 50 μm

**Fig. 2:**
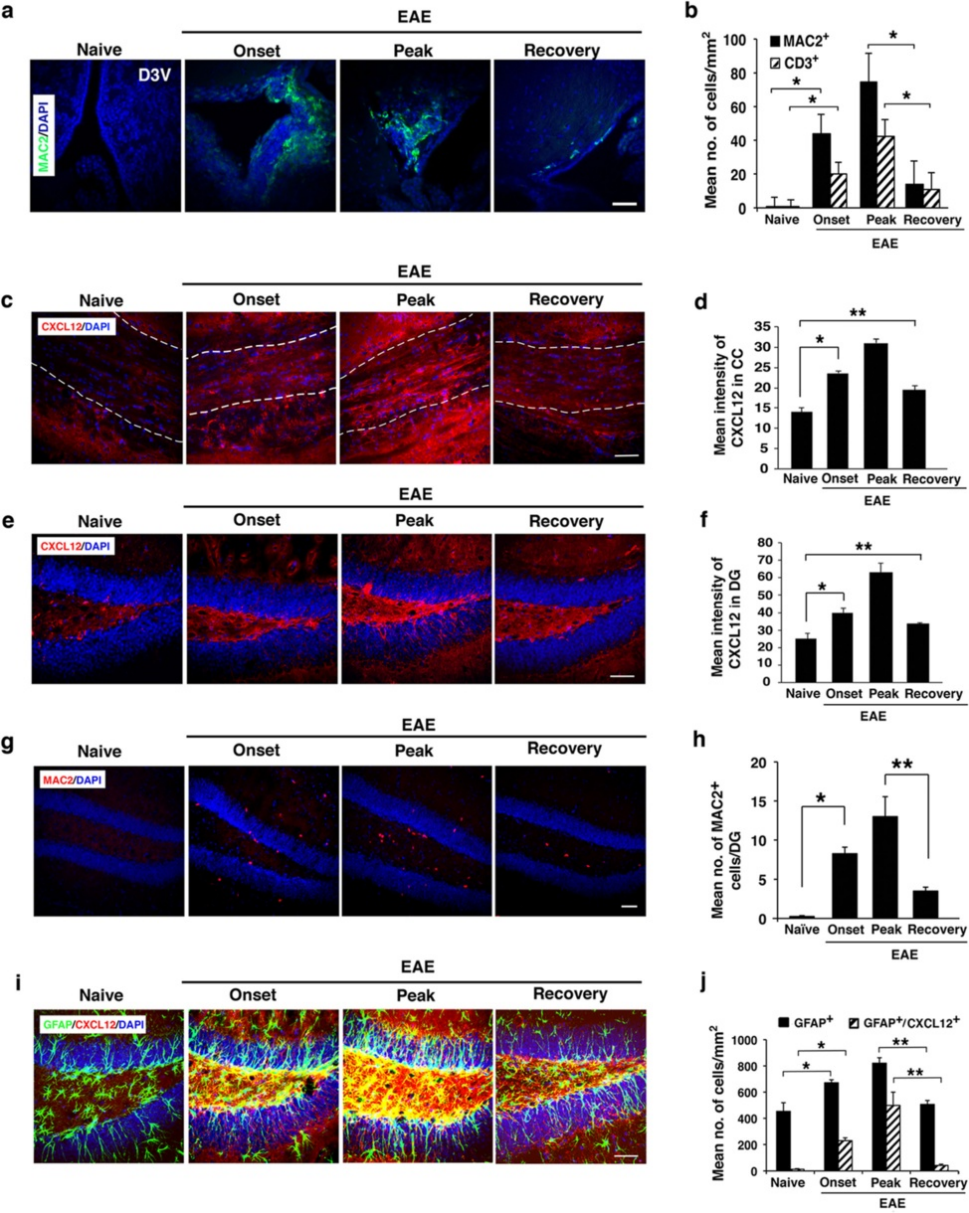
Expression of CXCL12 in the CNS of mice during progression of clinical EAE and following spontaneous recovery. **a**, **b** Immunostaining for MAC2 (*green*) in brain sections from the D3V and the quantification of activated macrophages/microglia (MAC2^+^) and infiltrating lymphocytes (CD3^+^) in brain tissue sections from naïve mice or mice at onset, peak, or following recovery (*p < 0.0001; CD3 representative immunostaining image not shown). **c**, **d** Immunostaining for CXCL12 (*red*) in brain sections from CC (central/caudal regions, denoted by *dashed lines*), and respective quantification of CXCL12 intensity (*p = 6.5 × 10^−5^, **p = 0.001). **e**, **f** Immunostaining for CXCL12 (*red*) in brain sections from the DG, and the respective quantification of CXCL12 intensity in brain sections from DG (*p = 0.004, **p = 0.039). **g**, **h** Immunostaining for MAC2 (*red*) in the DG and the respective quantification of activated macrophages/microglia (MAC2^+^) (*p = 0.0003, **p = 0.006). **i**, **j** Co-immunostaining for GFAP (*green*) and CXCL12 (*red*) in brain sections from the DG, **i** and the respective quantification of astrocytes (GFAP^+^) and astrocytes co-expressing CXCL12 (GFAP^+^ CXCL12^+^) in DG (*p ≤ 0.0002, **p ≤ 0.0001). Data in **b** and **d** are from nine consecutive sections from each mouse, *n* = 3 mice/group. Data in **f**, **h**, and **j** are from five consecutive sections from each mouse; *n* = 4 mice/group. *Error bars* represent mean ± SEM. All sections were counterstained with DAPI (*blue*) to visualize cell nuclei. Scale bars: **a**, **c**, **e**, **g**, and **i** 50 μm

Sites of inflammation in the CNS of mice with EAE are associated with increased levels of CXCL12 [3, 11, 13]. Accordingly, strong immunofluorescent staining of CXCL12 was detected in the posterior funiculus of the spinal cord, expressed mainly by GFAP^+^ astrocytes (Additional file 1: Figure S1a, b), and in the cortex (Additional file 1: Figure S1c) of mice with EAE. Unexpectedly, however, analysis of brains of mice at different stages of EAE (onset, peak, and recovery, as depicted in Fig. 1a) revealed that the level of CXCL12, which intensified during disease progression, remained high in the brains of mice that spontaneously recovered from clinical EAE (Fig. 1b, c; about twofold higher vs. naïve mice, *p* < 10^−9^; 7–10 days after mice were free of clinical manifestations) and returned to its basal level within 20 days after clinical recovery (data not shown). Notably, the levels of CXCL12 remained relatively high despite a decline in CNS inflammation (Fig. 2a, b; 5.3- and 3.9-fold decreases in MAC2^+^ and CD3^+^ cells, respectively, at recovery compared to peak; *p* < 0.0001).

The relatively high levels of CXCL12 that were sustained in the CNS after mice recovered, and CNS inflammation was abolished, raised the intriguing possibility that CXCL12 may play a beneficial physiological post-inflammatory role. This hypothesis was based on data indicating that CXCL12 promotes differentiation and survival of neurons [31] and neuron migration during CNS development [11, 12, 14], and on its effect on the differentiation/maturation of OPCs in vitro [27] or in vivo [8, 22]. To further explore this post-inflammatory function, we evaluated CXCL12 expression during disease progression and following recovery in the CC, and in the DG of the hippocampus as a known neurogenic niche. We observed similar dynamic expression patterns of CXCL12 in the CC and DG, at different stages of EAE and following recovery. There was a 2.2-fold increase in expression level of CXCL12 in the CC at the peak of disease compared to its expression in naïve mice (Fig. 2c, d). Following recovery, the expression of CXCL12 decreased only slightly to 62.8 % of its level at the peak of the disease and remained 1.38-fold higher than naïve levels (*p* < 0.001; Fig. 2d). Similarly, the level of CXCL12 in the DG increased with disease progression to about 2.5-fold of its basal level in DG of naïve mice at the peak of the disease; after spontaneous recovery, CXCL12 expression declined to 53.4 % of its level at the peak of the disease (*p <* 0.002 Figs. 2e, f) and remained 1.35-fold higher than naïve levels (*p* = 0.039; Fig. 2f). Thus, the CXCL12 levels remained elevated in CC and DG even after recovery when the numbers of infiltrating CD3^+^ T cells (data not shown) and MAC2^+^ inflammatory cells in DG were dramatically reduced (Fig. 2g, h). The persistence of CXCL12 in the DG after clinical and pathological recovery was observed in all mice tested, suggesting that CXCL12 may have a physiological post-inflammatory role, possibly in supporting neural repair.

We next sought to determine the source of the high levels of CXCL12 in the CNS of EAE-recovered mice, where only few inflammatory infiltrating cells remained after the CNS inflammation subsided. Astrocytes are believed to be the major source of CXCL12 in the CNS of MS patients [3] as well as in CNS of mice with cuprizone-induced demyelination [8]. To examine whether astrocytes were the source of CXCL12 present in the DG of EAE-recovered mice, we determined the number of astrocytes (GFAP^+^) as well as astrocytes expressing CXCL12 in the DG of mice during EAE progression and recovery. As shown in Fig. 2i, j, the numbers of GFAP^+^ CXCL12^+^ astrocytes were dramatically increased with disease progression both in the DG hilus and SGZ. However, by 7–10 days after clinical recovery, the numbers of GFAP^+^ CXCL12^+^ astrocytes declined almost to basal levels, whereas levels of CXCL12 were sustained (Figs. 2e, f). Thus, the astrocytes are not likely to be the major source of CXCL12 in the DG of EAE-recovered mice.

It should be noted that the immunostaining analysis of the DG and CC in mice that had not recovered spontaneously from EAE (non-recovered) (scarified for the analysis at the end point of the experiment on day 65 post-immunization, Fig. 1a) showed staining intensity for CXCL12, macrophages, and GFAP+ astrocytes that was higher than that in spontaneously recovered mice, albeit lower than that in mice at the peak of the disease. (Additional file 2: Figure S2).

### Numbers of DCX^+^ and DCX^+^ CXCL12^+^ NPCs increase with spontaneous recovery

**Fig. 3:**
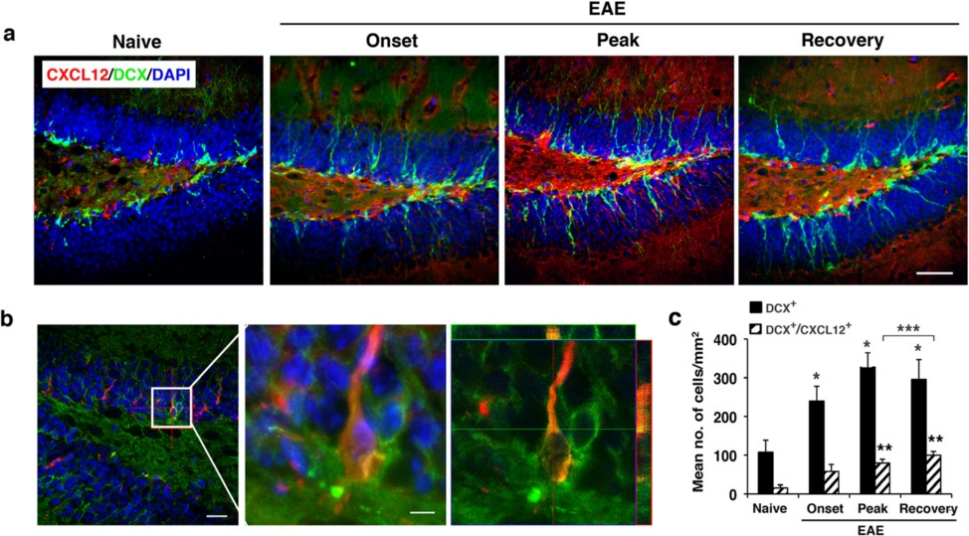
The numbers of NPCs and of CXCL12^+^ NPCs increase in DG of mice with the onset and the progression of clinical EAE and remain relatively high following spontaneous recovery. **a** Representative images of the DG of mice with EAE clinical episodes or following spontaneous recovery; sections were co-immunostained for DCX (*green*) and CXCL12 (*red*). **b** Orthogonal view of three-dimensional analysis showing NPC (DCX^+^, *green*) expressing CXCL12 (*red*). The middle image is a higher magnification of *boxed area* in the *left image* taken from DG of EAE-recovered mouse. Colocalization of DCX (*green*) and CXCL12 (*red*) was confirmed by optical dissection and orthogonal reconstruction of the confocal image (*right image*). **c** Quantitative analysis of the NPCs (DCX^+^) and NPCs co-expressing CXCL12 (DCX^+^ CXCL12^+^) in SGZ of the DG of mice with EAE clinical episodes or spontaneously recovered mice (*p < 7.4 × 10^−5^ and **p < 3.3 × 10^−7^, compared to naïve; ***p < 0.005, recovered compared to peak). Data are from five consecutive sections from each mouse, *n* = 3 mice/group. Data are the mean ± SEM from two independent experiments. Nuclei were visualized by DAPI counterstaining (*blue*). Scale bars: **a**, 50 μm; *left image* in **b**, 20 μm; *boxed area* in **b**, 5 μm

Despite the remarkably high levels of CXCL12 in the spinal cord (Additional file 1: Figure S1a, b), we did not detect CXCL12 expression in the relatively few NPCs found in the spinal cord of mice with EAE (data not shown). In contrast, a significant number of CXCL12-immunoreactive NPCs (DCX^+^ CXCL12^+^) were detected at the SGZ of the DG of mice during progression of EAE and, notably, also following recovery (Fig. 3a, c). In the immunostained sections from non-recovered mice (on day 65 post-immunization) DCX^+^ NPCs co-expressing CXCL12 were also noted, and the immunofluorescence intensity of DCX/CXCL12 co-staining was significantly lower than that of immunostained sections from recovered mice and was more comparable to immunostained sections of mice at the peak of the disease (Additional file 2: Figure S2e). The colocalization of CXCL12 with DCX immunostaining (Fig. 3b), which strongly suggests in situ expression of CXCL12 by the NPCs in the DG of mice with EAE, is in line with previous reports of NPCs expressing CXCL12^+^ [4, 32, 33]. Quantitative analysis of the hippocampal CXCL12 immunoreactive NPCs showed an approximately fivefold increase in DCX^+^ CXCL12^+^ NPCs at the peak of the disease compared to naïve mice (*p* < 3.3 × 10^−7^) (Fig. 3c). The total number of NPCs (DCX^+^) in the DG was also increased during EAE progression (approximately threefold; *p* < 7.4 × 10^−5^), albeit to a lesser extent than DCX^+^ CXCL12^+^ NPCs, compared to naïve mice (Fig. 3c). Remarkably, 7–10 days after the recovery from clinical symptoms of EAE, the number of the DCX^+^ CXCL12^+^ NPCs was increased even further in the SGZ of the DG (~1.3-fold above levels at peak of disease, *p* < 0.005; and ~6.4-fold above naïve mice, *p* < 3.3 × 10^−7^) (Fig. 3c), even though inflammation in the DG was minimal (Figs. 2g, h). These results suggest a potential involvement of CXCL12 in regulation of neuronal progenitor cell replenishment in the brain, both during the EAE-incurred CNS inflammation/damage, and even more so at the recovery stage, when inflammation is diminished.

### NG2^+^ and NG2^+^ CXCL12^+^ OPCs are significantly elevated in the CNS of EAE-recovered mice

**Fig. 4:**
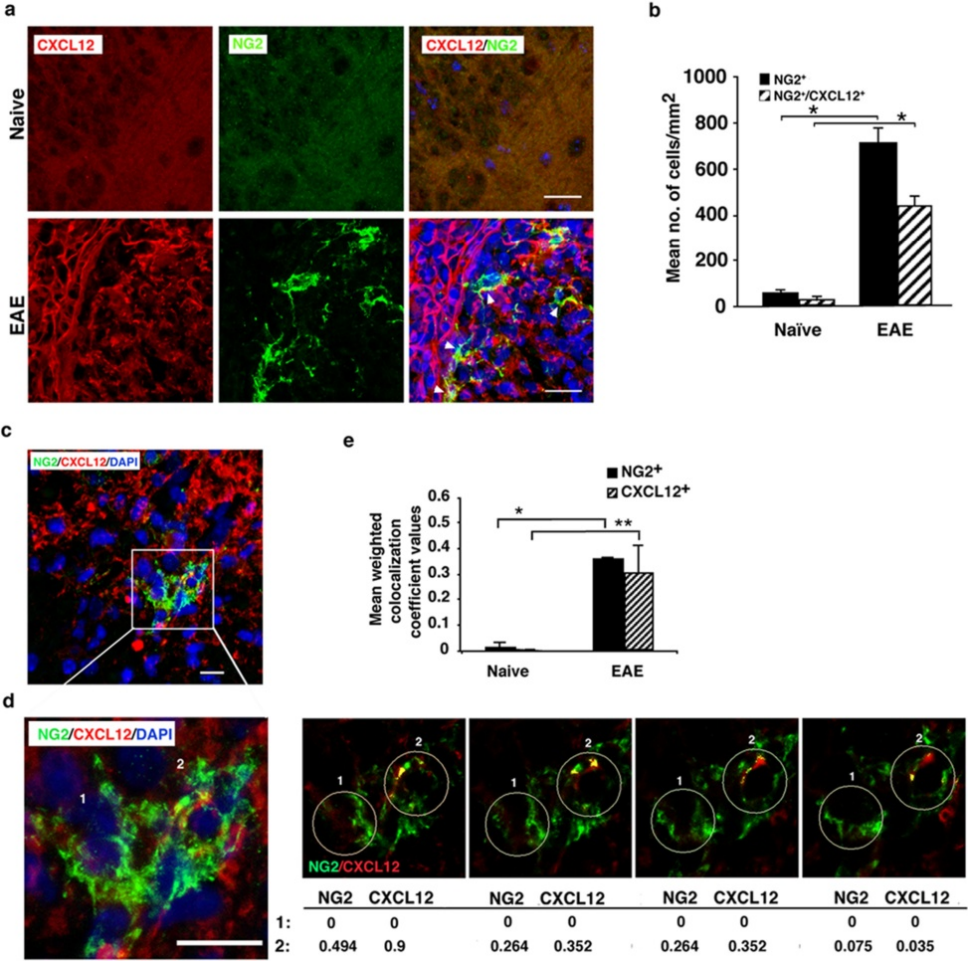
Numbers of CXCL12^+^ OPCs are elevated in the spinal cord of mice as EAE progresses and are further increased following spontaneous recovery from the disease. **a** Representative images of the spinal cord tissue sections from naïve healthy mice and from mice with clinical EAE (at the peak) co-immunostained for OPCs (NG2^+^, *green*) and CXCL12 (*red*). **b** Quantitative analysis of OPCs (NG2^+^) co-expressing CXCL12 (NG2^+^ CXCL12^+^) in the spinal cord of mice with EAE compared to naïve mice (*p < 10^−7^: data are from five consecutive sections per mouse, *n* = 3 mice/group). **c**–**e** NG2^+^ OPCs and CXCL12 colocalize in the spinal cord sections of mice with EAE. **c** Confocal image of two OPCs (NG2^+^, *green*): one is CXCL12 positive (*red*) and the other is CXCL12 negative, in a spinal cord section (*L4*) from a mouse with clinical EAE. **d** The *left image* is a higher magnification of the *boxed area* in **c**. The *right panel* represents colocalization analysis of serial levels (each 0.5 μm). *Numbers* in the table represent weighted colocalization coefficient (WCC) values of NG2^+^ cell that do not express CXCL12 (marked 1) compared to NG2^+^ cell that co-express CXCL12 (marked 2). WCC values were measured with Zeiss LSM image examiner software. In the table, values for CXCL12 (*red*) denote the colocalization coefficient from the *red channel* colocalized with the *green channel*, and the values for NG2 (*green*) denote the colocalization coefficient from the green channel colocalized with the red channel for OPCs marked 1 and 2. **e** Quantitative analysis of the mean WCC values of NG2^+^ OPCs co-expressing CXCL12 in mice with EAE compared to naïve mice (*p < 0.001, **p = 0.002). Data are from five consecutive sections from each mouse, *n* = 3 mice/group. *Error bars* represent mean ± SEM from two independent experiments. Nuclei were visualized by DAPI staining (*blue*). Scale bars: **a** 20 μm; **c**, **d** 10 μm. *Arrowheads* indicate OPCs (NG2^+^) that co-express CXCL12

**Fig. 5:**
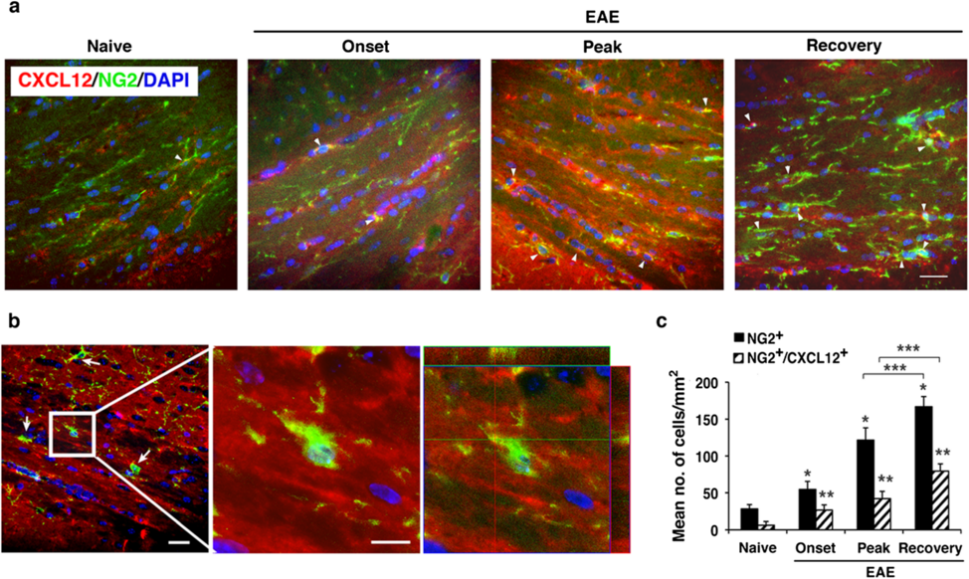
Numbers of CXCL12^+^ OPCs are elevated in the CC of mice with onset and the progression of clinical EAE and further increase following spontaneous recovery from the disease. **a** Representative images of the CC of mice with EAE clinical episodes or following spontaneous recovery; sections were co-immunostained for OPCs (NG2^+^, *green*) and CXCL12 (*red*). **b** Orthogonal view of an OPC (NG2^+^, *green*) in the CC of EAE-recovered mouse that express CXCL12 (*red*). The *middle image* is a higher magnification of the boxed area in the *left image*. Colocalization of NG2 (*green*) and CXCL12 (*red*) was confirmed by optical dissection and orthogonal reconstruction of the confocal image (*right image*). **c** Quantitative analysis of the OPCs (NG2^+^) and OPCs that co-express CXCL12 (NG2^+^CXCL12^+^) in the CC of mice with EAE clinical episodes or following spontaneous recovery (*p < 0.00001 and **p < 0.0003 compared to naive; ***p < 0.0005, recovered compared to peak, *n* = 4 mice/group). Data are from five consecutive sections from each mouse. Data represent mean ± SEM from three independent experiments. Nuclei were visualized by DAPI counterstaining (*blue*). Scale bar: **a** 50 μm; left image in **b**, 50 μm; boxed area in **b**, 5 μm. *Arrowheads* indicate OPCs (NG2^+^) co-expressing CXCL12

Consistent with previous studies [8, 34], we observed a dramatic increase in numbers of NG2^+^ OPCs in the spinal cord and in the CC (Figs. 4 and 5) of mice with EAE. Quantitative analysis showed a remarkable increase (by 11-fold; *p* ≤ 10^−7^) in the number of NG2^+^ OPCs in the spinal cords of mice with clinical EAE compared to naïve mice (Fig. 4a, b); of these, 61 % co-expressed CXCL12 (Fig. 4b). Unlike NPCs and mature neuronal cells that were shown in prior studies to express CXCL12 [4, 32, 33], the expression of CXCL12 by OPCs or oligodendrocytes had not been reported. To verify that the co-immunostaining of CXCL12 with NG2^+^ cells reflects the expression of CXCL12 by the OPCs rather than a result of their binding CXCL12 secreted by other cells such as activated microglial cells or astrocytes, we carried out a colocalization analysis at high magnification on sections of spinal cords from mice with EAE that were co-stained for NG2, CXCL12, and DAPI. Figure 4c shows a spinal cord section from mouse with EAE that includes two adjacent NG2^+^ cells: one is negative and the other one is immunoreactive for CXCL12. The cell marked 2 in Fig. 4c was double-labeled for NG2 and CXCL12; whereas the cell marked 1 was immunoreactive for NG2 but negative for CXCL12 in four consecutive confocal slices (Fig. 4d). The microscopic observations were confirmed by evaluating the weighted colocalization coefficients (WCC; see “Methods” section), which measured the pixel overlap between NG2 and CXCL12. Quantitative analysis of the mean WCCs in the spinal cord sections from mice with EAE indicated values of 0.360 ± 0.007 and 0.31 ± 0.13 for NG2 and CXCL12, respectively, compared to 0.014 ± 0.012 and 0.003 ± 0.004 in the spinal cord sections from naïve healthy mice (Fig. 4e), further indicating that the OPCs in the spinal cord of mice with EAE express CXCL12.

The numbers of OPCs and of CXCL12-expressing OPCs (NG2^+^ CXCL12^+^) in the CC were also increased with the progression of the disease and further increased following clinical recovery (Fig. 5). Figure 5a shows representative images of CC from mice at different stages of EAE co-immunostained for CXCL12, NG2, and DAPI. In the immunostained sections from non-recovered mice (on day 65 post-immunization) NG2^+^ OPCs co-expressing CXCL12 were also noted, and the immunofluorescence intensity of NG2/CXCL12 co-staining was significantly lower than that of immunostained sections from recovered mice and more comparable to immunostained sections of mice at the peak of the disease (Additional file 2: Figure S2f). The orthogonal view (Fig. 5b) showing colocalization of NG2^+^ and CXCL12^+^ suggests the presence of OPCs expressing CXCL12 also in the CC. Quantitative analysis of NG2^+^ and NG2^+^ CXCL12^+^ OPCs in the CC showed their significant increase at EAE onset and at disease peak compared to naïve mice (Fig. 5c). At the peak of the disease, the numbers of NG2^+^ and NG2^+^ CXCL12^+^ cells expanded by 4.3- (*p* < 10^−5^) and 6.7- (*p* < 3 × 10^−4^) fold, respectively, compared to naïve mice. Interestingly, the numbers of NG2^+^ and NG2^+^ CXCL12^+^ OPCs continued to expand by 5.9- (*p* < 10^−5^) and to 12.7- (*p* < 3 × 10^−4^) fold, respectively, in the CC of mice that recovered from clinical EAE compared to naïve mice. These results strongly suggest the involvement of CXCL12 also in the regulation of OPCs both in the CC and spinal cord.

### Expression of CXCL12 by in vitro differentiating OPCs and NPCs

**Fig. 6:**
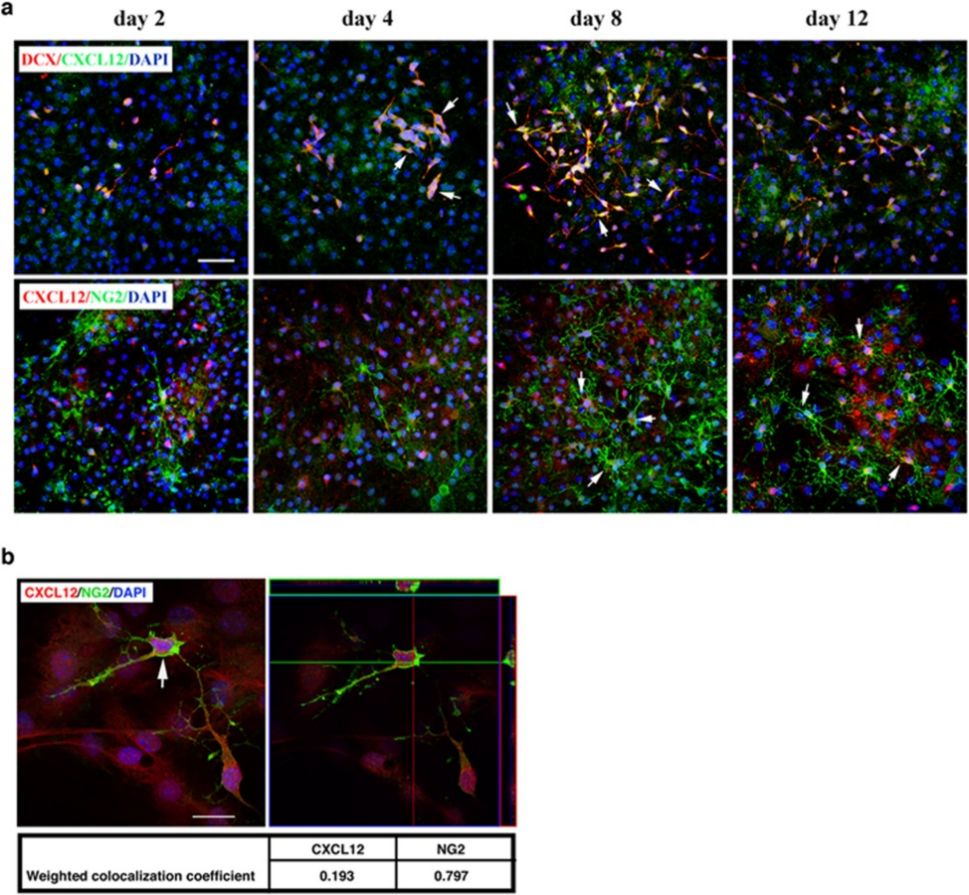
Expression of CXCL12 by NPCs and OPCs differentiated from aNSCs in vitro. **a** Representative images of differentiating aNSCs co-stained for DCX (*red*) and CXCL12 (*green*) (*upper panel*) or for NG2 (*green*) and CXCL12 (*red*) (*lower panel*) at the indicated time points of differentiation. **b** Orthogonal view from confocal *z*-series of OPC co-expressing CXCL12 (indicated with *arrow in left image*) presented as viewed in *x*-*z* (*upper*) and *y*-*z* (*right*) planes (right image). The WCC values presented in the table demonstrate the colocalization of CXCL12 with NG2. Images shown are representative of three independent experiments with similar results. Nuclei were counterstained with DAPI (*blue*). Scale bars: **a** 50 μm; **b** 20 μm

Astrocytes are a known source of CXCL12 in the CNS [2, 3, 35]. To further determine whether cells of the other neural lineages, particularly OPCs, also produce CXCL12, we analyzed neural progenitor cells of in vitro differentiated adult NSCs (aNSCs) (dissociated neurospheres) (Additional file 3: Figure S3). Cultures from different days of differentiation were extensively washed and subjected to immunofluorescent staining for CXCL12 and markers of the different neural lineage progenitor cells. As was observed previously (reviewed in [11]), the in vitro differentiated astrocytes (GFAP^+^) expressed high levels of CXCL12 (data not shown). The DCX^+^ neuronal progenitor cells also co-expressed CXCL12 (Fig. 6a, upper panel), in line with the studies of Tran et al. [32]. Importantly, although not previously observed, a significant proportion of NG2^+^ OPCs co-expressed CXCL12 (Fig. 6a, lower panel). A confocal reconstruction demonstrated that CXCL12 was expressed by aNSCs that differentiated to NG2^+^ OPCs, as CXCL12 staining could be detected in the NG2^+^ cell body as well as along its processes (Fig. 6b) and the WCC for this cell demonstrated overlapping pixels between NG2 and CXCL12.

### CXCL12 promotes differentiation of NPCs and OPC in vitro

**Fig. 7:**
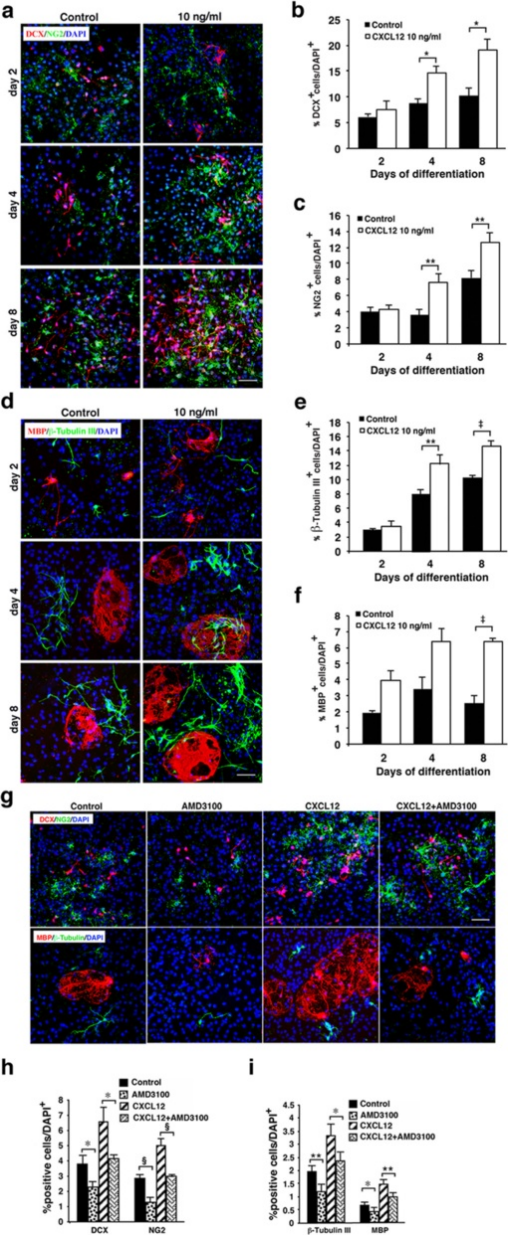
CXCL12 promotes and a CXCR4 antagonist inhibits differentiation and maturation of aNSCs. **a** Representative images of immunofluorescent staining for NPCs (DCX^+^, *red*) and OPCs (NG2^+^, *green*) in cultures of aNSCs undergoing differentiation in the absence or presence of CXCL12 (10 ng/ml). **b** Quantitative analysis of NPCs (DCX^+^). **c** Quantitative analysis of OPCs (NG2^+^). **d** Representative images of immunofluorescent staining for maturing neurons (βΙΙΙ-tubulin^+^, *green*) or oligodendrocytes (MBP^+^, *red*) in cultures of aNSCs undergoing differentiation in the absence or presence of CXCL12 (10 ng/ml). **e** Quantitative analysis of maturing neurons (βΙΙΙ-tubulin^+^). **f** Quantitative analysis of mature oligodendrocytes (MBP^+^). **g** Representative images of immunofluorescent staining (upper panel) for NPCs (DCX^+^, *red*) and OPCs (NG2^+^, *green*) and (*lower panel*) for maturing neurons (βΙΙΙ-tubulin^+^, *green*) or oligodendrocytes (MBP^+^, *red*) in cultures of aNSCs undergoing differentiation in the absence or presence of the CXCR4 antagonist, AMD3100 (100 ng/ml, added twice a day), CXCL12 (10 ng/ml) alone, or CXCL12 and AMD3100 combined after 5 days in culture. Cultures with AMD3100 alone or with no additive served as respective controls. **h** Quantitative analysis of neural progenitors NPCs (DCX^+^) and OPCs (NG2^+^). **i** Quantitative analysis of maturing neurons (βΙΙΙ-tubulin^+^) or oligodendrocytes (MBP^+^). Results in **b**, **c**, **e**, **f**, **h**, and **i** are presented as percentages of nuclei numbers and represent the mean ± SEM from three independent experiments. Nuclei were visualized by DAPI counterstaining. *p ≤ 0.05; **p = 0.02; ‡*p* ≤ 0.002; § *p* = 0.04. Scale bars: **a**, **d**, **g** 50 μm

Neurospheres were differentiated in the absence or presence of exogenous CXCL12 (5, 10, or 20 ng/ml) in differentiation medium. The effects of 10 and 20 ng/ml recombinant CXCL12 on the in vitro differentiation and maturation of aNSCs were higher than that of 5 ng/ml; effects of 10 and 20 ng/ml CXCL12 were quite comparable, with 10 ng/ml being somewhat more optimal (summarized in Additional file 4: Figure S4). Accordingly, only the effects of 10 ng/ml recombinant CXCL12 on differentiation and maturation of aNSCs (representative images and quantitation) are presented in Fig. 7. Exogenous CXCL12 (10 ng/ml) significantly enhanced the differentiation of aNSCs into NPCs (DCX^+^, Fig. 7a, b) and OPCs (NG2^+^, Fig. 7a, c). Enhanced differentiation was evident after a 4-day incubation in the presence of CXCL12 and was more pronounced after 8 days, with 2- and 1.5-fold increase in numbers of DCX+ and NG2+ cells, respectively (Fig. 7b, c). CXCL12 also promoted the maturation of neuronal cells and oligodendrocytes, as shown by immunostaining for β-tubulin III and myelin basic protein (MBP), markers of mature neuronal cells and oligodendrocytes, respectively (Fig. 7d–f). Quantitative analysis showed that maturation of neuronal cells (Fig. 7e) and oligodendrocytes (Fig. 7f) was significantly enhanced. Higher (20 ng/ml) and to a lesser extent also the lower (5 ng/ml) concentrations of CXCL12 had a similar effect (Additional file 4: Figure S4).

To further validate that CXCL12 promotes differentiation/maturation of NPCs and OPCs, the CXCR4 antagonist, AMD3100, was added to aNSCs cultured in differentiation medium in the absence or presence of CXCL12. After 5 days, AMD3100 strongly inhibited the differentiation of neuronal (DCX^+^) and oligodendrocyte (NG2^+^) progenitor cells (Fig. 7g, top panel) and of their maturation to neurons (β-tubulin III^+^) and oligodendrocytes (MBP^+^) (Fig. 7g, bottom panel). Quantitative analysis confirmed the significance of the effect of AMD3100 (Fig. 7h, i). Notably, the presence of AMD3100, in the absence of exogenous CXCL12, also inhibited differentiation and maturation of neuronal and oligodendrocyte progenitors (Fig. 7a, b), compared to control differentiation cultures (without AMD3100 or CXCL12). These results are in line with previous study that showed inhibition of CXCL12 activity by AMD3100 in vitro [36]. AMD3100 did not alter cell viability as confirmed by staining with Hoescht 33342 and propidium iodide (data not shown). The data obtained from this extensive in vitro analysis suggest the role of CXCL12 in directing the differentiation/maturation of neural progenitor cells in the CNS. Our results are consistent with those of previous studies showing that CXCL12 promotes, via CXCR4, the differentiation or maturation of OPCs or NPCs in vitro [27] and in vivo [8, 16].

### CXCR4 and CXCL12 are co-expressed by NPCs and OPCs

**Fig. 8:**
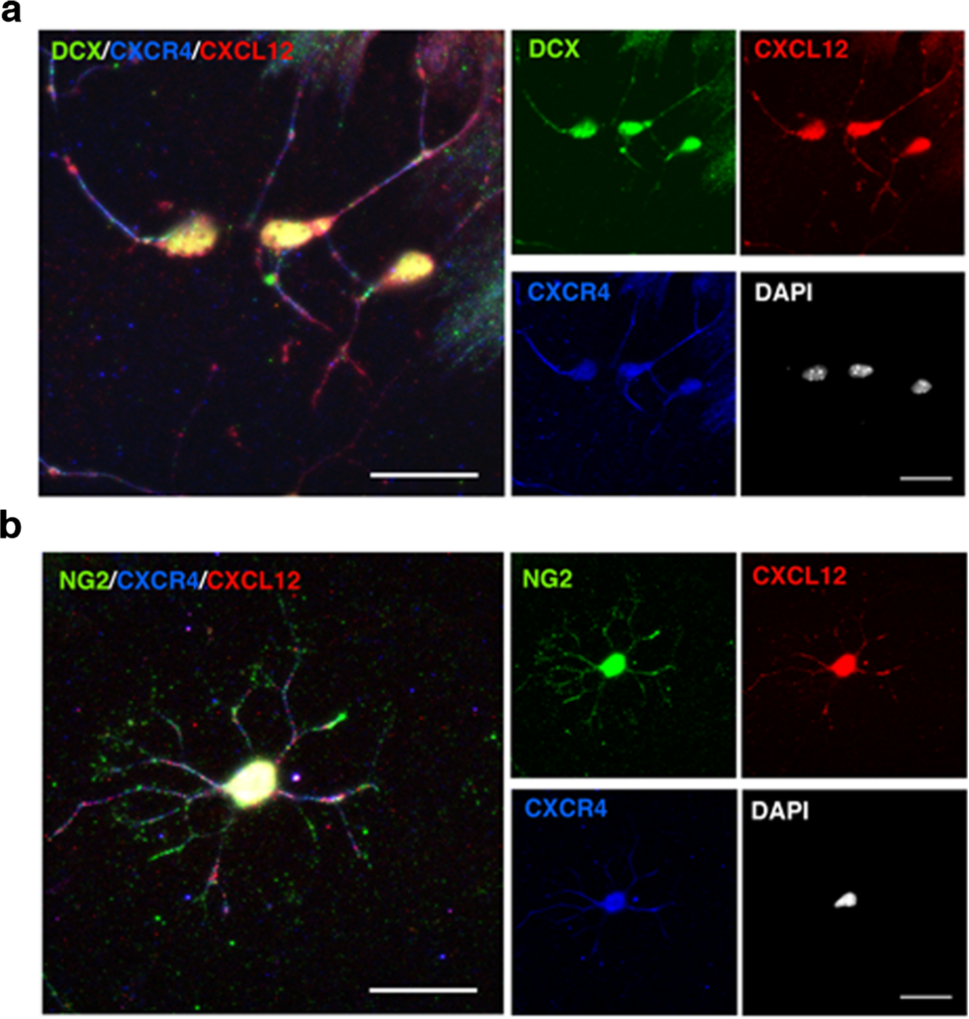
NPCs and OPCs differentiated in vitro co-express CXCR4 and its CXCL12 ligand. **a**, **b** Representative immunofluorescent images of **a** NPCs (DCX^+^, *green*) or **b** OPC (NG2^+^, *green*) co-labeled for CXCL12 (*red*) and CXCR4 (*blue*). Cells were differentiated from aNSCs cultured in differentiation medium for 6 days. Nuclei were visualized by DAPI counterstaining (*white*). Scale bar: 20 μm. The *panels at the right* show individual channels for NPCs or OPC co-stained for CXCR4 (*blue*) and CXCL12 (*red*). Scale bar: 20 μm

**Fig. 9:**
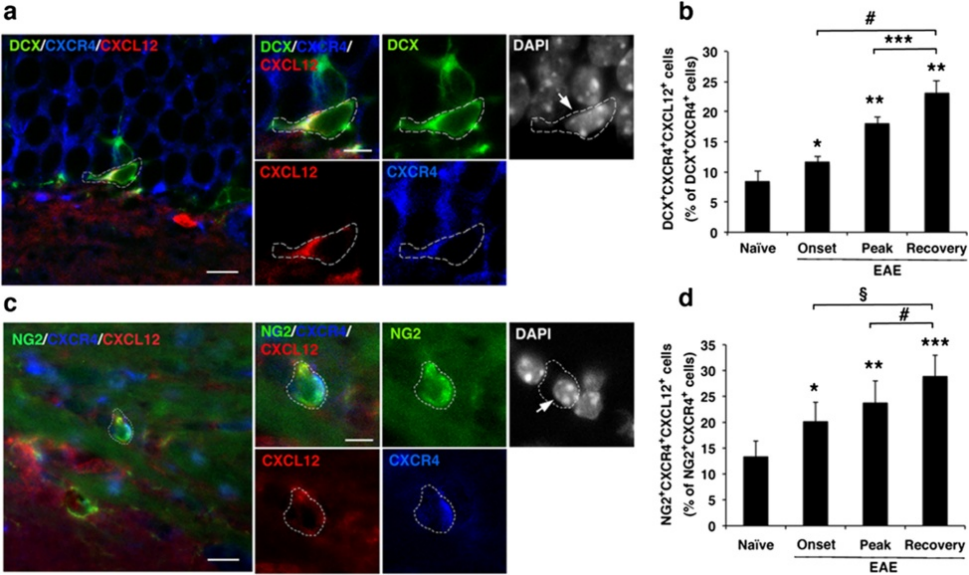
The proportion of neural progenitor cells co-expressing CXCR4 and CXCL12 is elevated with the onset and progression of clinical EAE and further increased following clinical recovery. **a** Representative immunofluorescent image of NPC in the DG of EAE-recovered mouse co-labeled for DCX (*green*), CXCR4 (*blue*), and CXCL12 (*red*). Cell marked with *dashed line* is shown in higher magnification in the panel on the right in individual channels and merged image. **b** Quantitative analysis of the percentage of NPCs (DCX^+^) that co-express CXCR4 and CXCL12 (DCX^+^ CXCR4^+^ CXCL12^+^) out of DCX^+^ CXCR4^+^ NPCs in the DG of mice with indicated EAE clinical episode or following spontaneous recovery. *p = 0.01 and **p < 4 × 10^−5^ compared to naïve; ***p = 0.004; #*p* = 6.5 × 10^−5^. **c** Immunofluorescent image of OPC in the CC of EAE-recovered mice co-labeled for NG2 (*green*), CXCR4 (*blue*), and CXCL12 (*red*). Cell marked with *dashed line* is shown in higher magnification in the panel on the right in individual channels and merged image. **d** Quantitative analysis of the percentage of OPCs co-expressing CXCR4 and CXCL12 (NG2^+^ CXCR4^+^ CXCL12^+^) out of NG2^+^ CXCR4^+^ OPCs in the CC of mice with indicated EAE clinical episode or following spontaneous recovery. Percentages were determined in five consecutive sections from each mouse (*n* = 3). Data are mean ± SEM from two independent experiments. *p = 0.01, **p = 0.003, and ***p = 0.0002 compared to naïve; #*p* = 0.04 (one tail *t* test); §*p* = 0.004. Nuclei were visualized by DAPI counterstaining (*white*). Scale bar: **a**, **c** 10 μm; *bar in right images* 5 μm

CXCR4 is expressed by mature neuronal cells [37, 38], NPCs [32], and OPCs [8, 26, 27, 39]; therefore, our finding that CXCL12 is expressed by NPCs and by OPCs suggests the intriguing possibility that neural progenitor cells may in fact express both the CXCR4 receptor and its CXCL12 ligand. To confirm co-expression of CXCR4 and CXCL12 by NPCs and particularly by the OPCs, in vitro differentiated cultures of aNSCs were co-stained for DCX/CXCR4/CXCL12 or for NG2/CXCR4/CXCL12. As shown in Fig. 8, a significant proportion of NPCs (Fig. 8a) and OPCs (Fig. 8b) in these cultures co-expressed CXCR4 and CXCL12. The frequencies of DCX^+^ CXCR4^+^ CXCL12^+^ among DCX^+^ NPCs and of NG2^+^ CXCR4^+^ CXCL12^+^ among NG2^+^ OPCs in the dense differentiation cultures were relatively high (>50 %, data not shown). The finding that NPCs and OPCs co-express the CXCR4 receptor and its CXCL12 ligand suggested that differentiation/maturation of these neural cells may occur via autocrine signaling mechanism [40, 41]. It was, therefore, of high significance to examine whether neural progenitor cells co-expressing CXCR4 and CXCL12 could be detected also in the CNS. This analysis was carried out in the CNS of mice with ongoing EAE or following spontaneous recovery as more CXCL12^+^ NPCs and CXCL12^+^ OPCs were detected in the CNS of these mice compared to naïve mice (Figs. 3, 4, and 5). Figure 9a, c shows representative images of a DCX^+^ NPC and an NG2^+^ OPC from EAE-recovered DG and CC, respectively, which co-express CXCR4 and CXCL12. Quantitative analysis revealed that the frequency of NPCs that co-express CXCR4 and CXCL12 among DCX^+^ CXCR4^+^ NPCs, and of OPCs that co-express CXCR4 and CXCL12, among NG2^+^ CXCR4^+^ OPCs, were over 20 % in EAE-recovered mice (Fig. 9b, d). Strikingly, a fraction of the DCX^+^ CXCR4^+^ NPCs and NG2^+^ CXCR4^+^ OPCs in naïve mice also co-expressed CXCL12 (Fig. 9b, d).

In mice developing EAE, the proportion of DCX^+^ NPCs that co-expressed CXCR4 and CXCL12 (out of DCX^+^ CXCR4^+^ NPCs in DG) increased significantly at EAE onset to 11.59 ± 0.90 % (*p* = 0.01), and to 18.0 ± 1.0 % (*p* < 4 × 10^−5^) at the peak of disease, compared to 8.37 ± 1.80 % in naïve mice) (Fig. 9b). Moreover, following clinical recovery from the disease, the proportion of DCX^+^ CXCR4^+^ CXCL12^+^ NPCs further increased to 23.00 ± 2.13 % (~2.7-fold increase compared to naïve mice, *p* < 4 × 10^−5^ and 1.98-fold compared to mice with ongoing EAE, *p* = 6.5 × 10^−5^). In parallel, the frequency of NG2^+^ CXCR4^+^ CXCL12^+^ OPCs among the NG2^+^ CXCR4^+^ OPCs in the CC significantly increased to 20.0 ± 3.7 % at EAE onset and to 23.7 ± 4.3 % at the peak of the disease, compared to 13.31 ± 3.05 % in naïve CC (*p* = 0.01 and *p* = 0.003, respectively) (Fig. 9d). Furthermore, as shown in Fig. 9d, the percentage of NG2^+^ CXCR4^+^ CXCL12^+^ OPCs among the NG2^+^ CXCR4^+^ OPCs in the CC further increased following clinical recovery to 28.80 ± 4.12 %, 2.16-fold over naïve mice (*p* = 0.0002) and 1.43-fold over mice with ongoing EAE (*p* = 0.004). Hence, the proportion of OPCs and NPCs in the CNS that co-expressed CXCR4 and CXCL12 increased as disease progressed and were further elevated during recovery, even though CNS inflammation has subsided. These findings further suggest the involvement of CXCL12 in the endogenous myelin/neuronal repair of the EAE-associated CNS damage by increasing endogenous neuro/oligodendrogenesis and possibly also the differentiation of NPCs and OPCs.

These increased numbers of CXCR4^+^ CXCL12^+^ neural progenitor cells following recovery are of high significance as a self-sufficient myelin/neural repair mechanism (autocrine signaling) in the absence of CXCL12 after the inflammatory cells diminished.

## Discussion

The data presented in this study highlight the post-inflammation role of CXCL12 in the context of endogenous myelin/neuronal repair associated with the spontaneous recovery from chronic clinical EAE. The association of CXCL12 with a potential repair of EAE-associated CNS tissue damage was initially suggested by the unexpected but consistent detection of about twofold increase in the expression of CXCL12 in the CNS of mice that had spontaneously recovered from clinical EAE (compared to naïve mice). We observed a relatively high expression of CXCL12 in the CC and in the neurogenic DG of recovered mice up to 20 days after these mice were free of clinical symptoms and their CNS inflammation had resolved. Post-inflammatory expression of CXCL12 was associated with an increase in numbers of CXCL12^+^ NG2^+^ OPCs and CXCL12^+^ DCX^+^ NPCs in the CC and DG, respectively, of EAE-recovered mice not only compared to naïve mice but also relative to EAE-affected mice at the peak of the disease. These findings suggest the involvement of CXCL12 in the endogenous myelin/neuronal repair of the EAE-associated CNS damage is by increasing endogenous neuro/oligodendrogenesis and possibly also the differentiation of NPCs and OPCs. Prior in vitro studies [13, 42] and previous reports of the various functions of CXCL12 during CNS development and following injuries to the adult CNS, including demyelination models, suggest the effect of CXCL12 on the proliferation of neural progenitor cells [8, 10, 27, 43].

Strikingly, in the context of the beneficial post-inflammatory role of CXCL12, we found that in mice with EAE, a significant proportion of neural progenitor cells co-express both CXCL12 and its CXCR4 receptor (Fig. 9). Moreover, the proportions of these CXCL12^+^ CXCR4^+^ DCX^+^ NPCs and CXCL12^+^ CXCR4^+^ NG2^+^ OPCs, which increased with disease progression further increased in recovered mice (2.12- and 2.75-fold, respectively, compared to naïve mice; *p* ≤ 0.0002). These findings are consistent with potential endogenous neuro/oligodendrogenesis and neural differentiation that can contribute to myelin/neural repair via autocrine signaling mechanisms, independently of inflammation-induced CXCL12-producing cells (activated microglia/macrophages and astrocytes) which are cleared from the CNS during spontaneous recovery from clinical EAE.

The expression patterns and potential functions of CXCR4 and its ligand CXCL12 have been widely investigated during CNS development and in healthy or injured adult CNS [7, 10, 11, 21, 44]. However, few studies have analyzed the potential function of CXCL12 in the context of myelin or neuronal repair in CNS inflammatory and demyelinating diseases, such as EAE/MS [7, 8, 22, 25]. Our analysis of the expression dynamics of CXCL12 with progression of EAE and following recovery consistently indicate that CXCL12 expression is greater in the DG and CC of mice which spontaneously recovered from severe clinical EAE than in naïve mice. This was somewhat surprising since the activated macrophages/microglial cells and astrocytes, which were reported to be as the major source of CXCL12 in the inflamed CNS of MS patients [3, 11, 45] and in the cuprizone-induced model of demyelination [8, 9, 46] are abolished during the EAE recovery phase. Such an observation, therefore, raised the question not only of the potential function for elevated levels of CXCL12 in the DG and CC of EAE-recovered mice but also of the origin of such elevated levels of CXCL12 at the DG and the CC of EAE-recovered mice.

Our immunohistochemistry analysis suggested that NPCs and OPCs that co-express CXCL12 could also be the source of the CXCL12 that was detected at the DG and CC of EAE-recovered mice. The expression of CXCL12 by neuronal progenitors or mature neurons has been reported [33, 38, 47]. In contrast, the expression of CXCL12 by OPCs or by oligodendrocytes had not been described before either during development or in the adult CNS, despite intensive studies on the involvement of CXCL12 in the proliferation [9, 27, 39], differentiation, and maturation of OPCs [8, 27] and their CNS migration [27, 39]. We have, therefore, confirmed that NG2+ OPCs in the CC and the spinal cord of EAE-recovered mice do express CXCL12, using confocal three-dimensional reconstruction and colocalization analyses. A similar confocal microscopy analyses confirmed that also in vitro differentiating NG2^+^ OPCs co-express CXCL12, independent of inflammatory factors present in the CNS during active EAE, suggesting a vital function for a basal expression of CXCL12 by OPCs.

Numerous studies have reported the expression of the CXCR4 and CXCR7 receptors by NSCs, OPCs, and NPCs, and the functional responses to activation by their only ligand, CXCL12, during development [48] and in inflamed adult CNS, particularly in MS/EAE [7, 8, 11, 22, 49]. While our detection of CXCR4 expression by NG2^+^ OPCs and DCX^+^ NPCs in CC and DG, respectively, of EAE-recovered mice was not surprising in view of these studies and of similar observations for in vitro differentiated NPCs and OPCs [8, 11, 27, 36, 39] (and Figs. 8 and 9), the finding that a significant proportion of these CXCR4^+^ DCX^+^ NPCs and CXCR4^+^ NG2^+^ OPCs co-expressed also the CXCR4 ligand, CXCL12, was quite unexpected. Most importantly, the frequencies of CXCL12^+^ CXCR4^+^ DCX^+^ NPCs and CXCL12^+^ CXCR4^+^ NG2^+^ OPCs in the DG and CC not only increased during disease progression but also during recovery with numbers significantly greater by about 1.5-fold than those at disease peak. These findings further support potential involvement of CXCL12 in promoting endogenous neuro/oligodendrogenesis and the differentiation/maturation of NPCs and OPCs towards myelin and neuronal repair following EAE-associated CNS tissue damage.

NG2^+^ OPCs and DCX^+^ NPCs that co-express CXCR4, and its ligand, CXCL12, have not been previously reported in EAE models or in patients with MS. Whether such CXCL12^+^ CXCR4^+^ neural progenitor cells can be intrinsically activated to differentiate via autocrine signaling mechanisms in MS/EAE has not been determined. Published data, however, suggest that CXCL12 can act as an autocrine modulator of neuronal activity [40, 47, 50]. Thus, it is plausible that the elevated numbers of CXCL12^+^ CXCR4^+^ DCX^+^ NPCs and of CXCL12^+^ CXCR4^+^ NG2^+^ OPCs that persist in the CNS of EAE-recovered mice are intrinsically activated via autocrine signaling mechanisms to differentiate into neural cells that would eventually mediate the repair of inflammation-associated myelin/neuronal damage. Such a self-sufficient mechanism (autocrine signaling) of NPCs and OPCs activation is likely to be essential for continuing the repair of the myelin/neural damage after the deleterious inflammation has subsided, and with it, the CXCL12 secreted by the inflammatory cells, particularly, when the small amounts of CXCL12 secreted by the vascular endothelia cells may not be sufficient for the intensive myelin/neuronal repair required after the deleterious inflammation.

We first detected an increase in the number of NPCs and OPCs at early clinical onset, during early inflammatory phase when the number of GFAP^+^ CXCL12^+^ astrocytes and the level CXCL12 also increase in the CNS ([2–4, 35], and this study). This suggests that the CXCL12, a major inflammatory cell chemoattractant [11], both facilitates inflammatory damage to CNS tissue during disease progression and promotes proliferation of NPCs and OPCs via paracrine signaling mechanisms, as many of the NPCs and OPCs express the CXCL12 receptor, CXCR4 [7, 8, 11, 27, 36]. The significant increase in neural progenitors during early EAE onset suggests that the endogenous neural repair mechanisms are being activated and built up already with development of the inflammation in CNS, and that the CXCR4-dependent activation by CXCL12 secreted by inflammatory cells may further drive the NPCs and OPCs towards differentiation/maturation and remyelination and neuronal repair [8, 10, 16]. Apparently, such myelin/neuronal protection/repair mechanisms are not sufficient to compete with the CNS damage incurred by inflammation. However, the endogenous repair process becomes more effective during the recovery phase when the CNS inflammation/damage is arrested and the CXCL12-expressing neural progenitors can increase in numbers and differentiate to mature neurons and oligodendrocytes without being perturbed by the deleterious inflammation.

The studies by McCandless et al. [4] and Meiron et al. [51] support the role of CXCL12 in neural protection following CNS inflammation in EAE. They showed in C57BL/6 mice that blocking CXCR4 activation by administering the CXCR4 antagonist, AMD3100 [4], or by anti-CXCL12 antibody [51] significantly increases the clinical severity of EAE induced by myelin oligodendrocyte glycoprotein which, unlike untreated mice, reach a plateau phase without significant remission. Different mechanisms were suggested in theses studies: aggravation of disease in the AMD3100-treated mice was attributed to elevated CNS inflammation associated with the disruption of CXCR4’s function in limiting the infiltration of autoreactive effector cells and migration of leukocytes into the parenchyma [4]. The disease aggravation by anti-CXCL12 antibodies was attributed to the role of CXCL12 in redirecting the polarization of effector Th1 cells into antigen-specific regulatory T cells in a CXCR4-dependent manner. However, the increase in the number of CXCR4^+^ NPCs and OPCs and of CXCL12 expressing CXCR4^+^ NPCs/OPCs already at early disease onset (shown above) suggests that the blockade of the CXCR4-dependent endogenous potential for neural protection/repair of CXCR4-expressing NPCs and OPCs through paracrine or autocrine CXCL12/CXCR4 signaling mechanisms may also be at play in disease aggravation and lack of remission following treatment with these agents. Therefore, it is likely that blocking the CXCR4-dependent promotion of NPC and OPC proliferation at an early phase of the disease can also contribute to aggravation of the disease by perturbing the remission mechanisms (myelin/neuronal protection) following treatment with AMD3100 or with anti-CXCL12 antibodies.

## Conclusions

This study highlights the post-inflammatory beneficial role of CXCL12 in adult CNS following MS-like inflammatory damage. In mice with EAE, the CXCL12/CXCR4 axis appears to be involved in promoting the endogenous myelin and neuronal repair capacity, particularly in mice undergoing spontaneous recovery. The spontaneous recovery from severe clinical EAE was associated with elevated CXCL12 expression in the DG and CC of spontaneously recovered mice, even though the CNS inflammation has subsided. The data presented above link CXCL12 expression in the DG and CC of EAE-recovering mice to promotion of the endogenous neuro/oligodendrogenesis. A significant proportion of the newly generated progenitor cells are CXCR4^+^ CXCL12^+^ NPCs and OPCs, endowed with intrinsic potential of neuro/oligodendroglial differentiation and myelin/neuronal repair via autocrine signaling mechanisms. The detection of CXCL12^+^ CXCR4^+^ DCX^+^ NPCs and CXCL12^+^ CXCR4^+^ NG2^+^ OPCs in the DG and CC, respectively, of mice with EAE and their significant increase in numbers in spontaneously recovered mice are consistent with a post-CNS-inflammation role of CXCL12 in promoting the endogenous potential of repairing myelin and neuronal damage, likely via self-sufficient (autocrine) signaling mechanism which is not dependent on CXCL12 secreted during inflammation.

Notably, the detection at the DG and CC of naïve mice of relatively small, but consistent, numbers of CXCL12^+^ CXCR4^+^ NPCs and CXCL12^+^ CXCR4^+^ OPCs, respectively, may also link the CXCL12/CXCR4 axis to the maintenance of neural homeostasis in normal adult CNS through autocrine signaling mechanisms. This intriguing possibility warrants further investigation.

## Additional files

Additional file 1: Figure S1. High expression of CXCL12 in the CNS of mice with chronic clinical EAE. (a, b) Immunofluorescent images of spinal cord tissue sections (L1–L6) from mice with ongoing EAE (day 50 after immunization; clinical score =3) and from naive mice (a) immunostained for CXCL12 (red) or (b) co-stained for CXCL12 (green) and GFAP (red). (c) Immunofluorescent image of the cortex of the mice in panel a immunostained for CXCL12 (red). In a and c, nuclei were counterstained with DAPI (blue). Scale bars: a and c, 100 μm; b, 20 μm. (TIF 8873 kb) Additional file 2: Figure S2. The CXCL12 expression, CXCL12^+^ NPCs and CXCL12^+^ OPCs in the DG and CC of mice that did not recover from EAE, compared to recovered mice or mice at the peak of the disease. Brain sections of mice that did not recover from EAE (non-recovered; clinical score 2) were taken at the end point of the experiment (day 65 post-immunization which is also close to the time points after immunization when EAE-recovered mice were taken for analysis; Fig. 1a). The sections were immmunostained for MAC2 (green) or CXCL12 (red), or for co-immunostained for CXCL12 and immune markers of astrocytes (GFAP), NPCs (DCX), or OPCs (NG2). The representative images from the immunostained sections from the non-recovered mice (*n* = 4) were placed for comparison side by side with the images from recovered mice, mice at the peak of the disease, and from naïve mice, which are presented in Fig. 2. Representative images show (a) immunostaining for MAC2 (green) in brain sections from the D3V. (b) Immunostaining for CXCL12 (red) in brain sections from CC (central/caudal regions, denoted by dashed lines). (c) Immunostaining for CXCL12 (red) in brain sections from the DG. (d) Co-immunostaining for GFAP (green) and CXCL12 (red) in brain sections from the DG. (e) Co-immunostaining for NPCs (DCX^+^, green) and CXCL12 (red) in the DG. (f) Co-immunostaining for OPCS (NG2^+^, green) and CXL12 (red) in CC. Nuclei were visualized by DAPI counterstaining (blue). The brain sections were from same immunizations. The images shown for the non-recovered mice were from immunostaining of preserved free-floating brain sections that were kept in the presence of NaN_3_ for 18 months. Scale bar: a–f, 50 μm. Arrowheads in f indicate OPCs (NG2^+^) co-expressing CXCL12. (TIF 9358 kb) Additional file 3: Figure S3. In vitro differentiation of aNSCs isolated from the subventricular zone of adult mice. Neurospheres that were expanded in neurosphere medium in the presence of growth factors were differentiated on PDL-coated coverslips in the presence of 5 % FCS in the absence of growth factors. On day 0, a very small proportion of cells in neurospheres were NPCs (DCX^+^), but none were OPCs (NG2^+^), maturing neuronal cells (NF200^+^), or oligodendrocytes (MBP^+^). The differentiation cultures were fixed after 2, 4, and 8 days and immunostained for indicated markers of neuronal, oligodendroglial, or astrocytic lineages. At day 2 of culture, differentiation to NPCs (DCX^+^), OPCs (NG2^+^), and astrocytes (GFAP^+^) were observed, which matured with time (day 4 and 8) to neuronal cells (β-tubulin III^+^), MBP-producing oligodendrocytes, or to astrocytes. Nuclei were visualized by DAPI counterstaining. Scale bar: 50 μm. (TIF 8316 kb) Additional file 4: Figure S4. CXCL12 promotes differentiation and maturation of aNSCs dose response. The data are from immunofluorescent staining for NPCs (DCX^+^, red) and OPCs (NG2^+^, green) (as in Fig. 7a) and for maturing neurons (βΙΙΙ-tubulin^+^, green) or oligodendrocytes (MBP^+^, red) (as in Fig.7d) of cultures of aNSCs undergoing differentiation in the absence or presence of CXCL12 (5, 10, or 20 ng/ml). The representative images for the effect of 10 ng/ml CXCL12, as an optimal dose, is shown in Fig. 7. The effects of the different concentration of CXCL12 on the in vitro differentiation and maturation of aNSCs are summarized here quantitatively. (a) Quantitative analysis of NPCs (DCX^+^). (b) Quantitative analysis of OPCs (NG2^+^). (c) Quantitative analysis of maturing neurons (βΙΙΙ-tubulin^+^). (d) Quantitative analysis of mature oligodendrocytes (MBP^+^). The effect of 5 ng/ml CXCL12 was lower than other concentrations; effects of 10 and 20 ng/ml CXCL12 were quite comparable, with 10 ng/ml being somewhat more optimal. We therefore showed in the body of the manuscript (Fig. 7) only the effects of CXCL12 at 10 ng/ml concentration. Results in a–d are presented as percentages of nuclei numbers and represent the mean ± SEM from three independent experiments. **p* ≤ 0.05; ***p* < 0.01. (TIF 8921 kb)

## Abbreviations

aNSCs: adult neural stem cells
CC: corpus callosum
DG: dentate gyrus
NPCs: neuronal progenitor cells
NSCs: neural stem cells
OPCs: oligodendrocyte precursor cells
PLP: proteolipid protein peptide
WCC: weighted colocalization coefficient
