# Supplementary figures and images for: Post-CNS-inflammation expression of CXCL12 promotes the endogenous myelin/neuronal repair capacity following spontaneous recovery from multiple sclerosis-like disease
Source: J Neuroinflammation. 2016 Jan 8;13:7. doi: 10.1186/s12974-015-0468-4 (PMC4706716; doi:10.1186/s12974-015-0468-4)

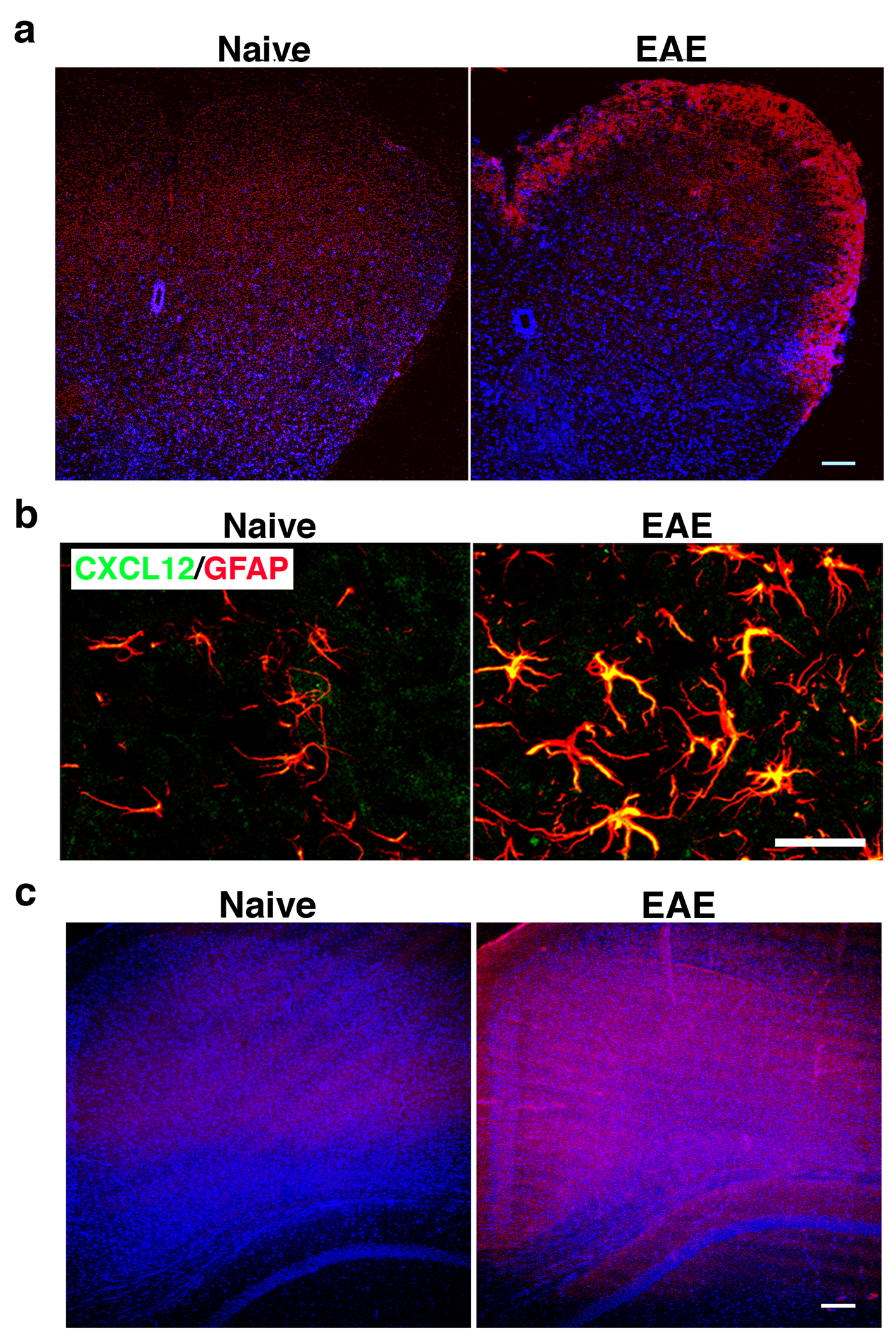

Supplement: Additional file 1: Figure S1. — High expression of CXCL12 in the CNS of mice with chronic clinical EAE. (a, b) Immunofluorescent images of spinal cord tissue sections (L1–L6) from mice with ongoing EAE (day 50 after immunization; clinical score =3) and from naive mice (a) immunostained for CXCL12 (red) or (b) co-stained for CXCL12 (green) and GFAP (red). (c) Immunofluorescent image of the cortex of the mice in panel a immunostained for CXCL12 (red). In a and c, nuclei were counterstained with DAPI (blue). Scale bars: a and c, 100 μm; b, 20 μm. (TIF 8873 kb) [file 12974_2015_468_MOESM1_ESM.tif]

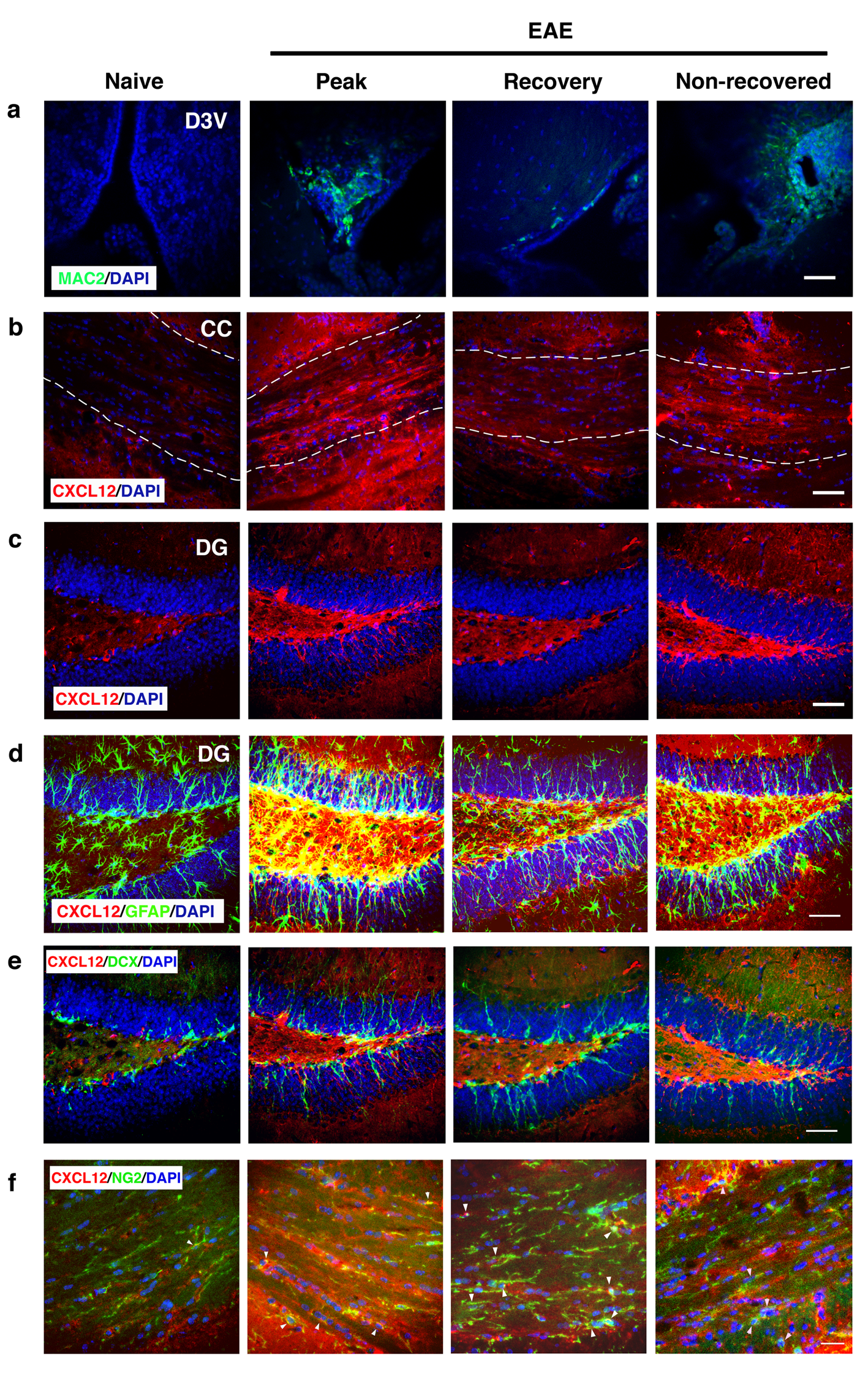

Supplement: Additional file 2: Figure S2. — The CXCL12 expression, CXCL12+ NPCs and CXCL12+ OPCs in the DG and CC of mice that did not recover from EAE, compared to recovered mice or mice at the peak of the disease. Brain sections of mice that did not recover from EAE (non-recovered; clinical score 2) were taken at the end point of the experiment (day 65 post-immunization which is also close to the time points after immunization when EAE-recovered mice were taken for analysis; Fig. 1a). The sections were immmunostained for MAC2 (green) or CXCL12 (red), or for co-immunostained for CXCL12 and immune markers of astrocytes (GFAP), NPCs (DCX), or OPCs (NG2). The representative images from the immunostained sections from the non-recovered mice (n = 4) were placed for comparison side by side with the images from recovered mice, mice at the peak of the disease, and from naïve mice, which are presented in Fig. 2. Representative images show (a) immunostaining for MAC2 (green) in brain sections from the D3V. (b) Immunostaining for CXCL12 (red) in brain sections from CC (central/caudal regions, denoted by dashed lines). (c) Immunostaining for CXCL12 (red) in brain sections from the DG. (d) Co-immunostaining for GFAP (green) and CXCL12 (red) in brain sections from the DG. (e) Co-immunostaining for NPCs (DCX+, green) and CXCL12 (red) in the DG. (f) Co-immunostaining for OPCS (NG2+, green) and CXL12 (red) in CC. Nuclei were visualized by DAPI counterstaining (blue). The brain sections were from same immunizations. The images shown for the non-recovered mice were from immunostaining of preserved free-floating brain sections that were kept in the presence of NaN3 for 18 months. Scale bar: a–f, 50 μm. Arrowheads in f indicate OPCs (NG2+) co-expressing CXCL12. (TIF 9358 kb) [file 12974_2015_468_MOESM2_ESM.tif]

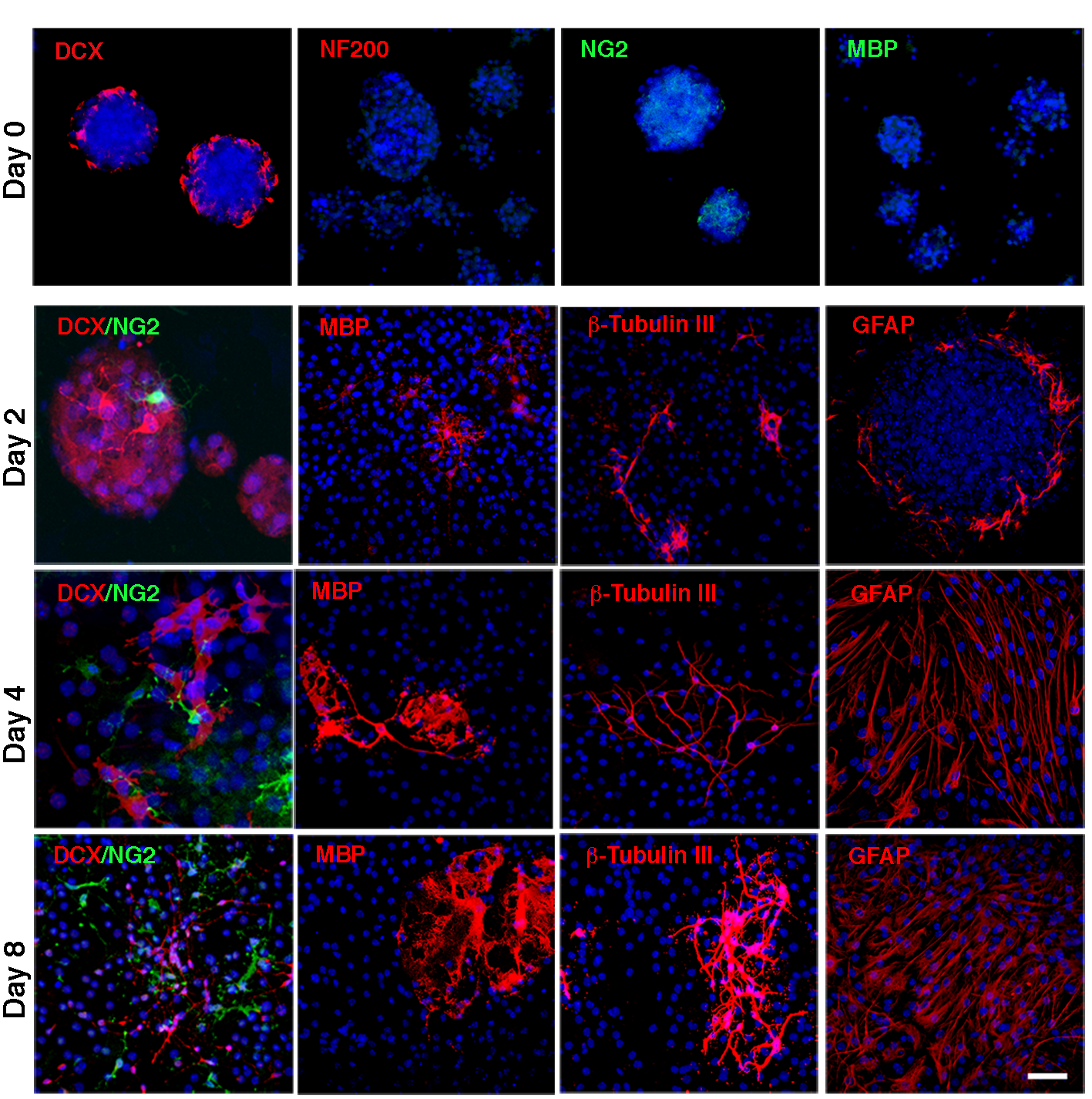

Supplement: Additional file 3: Figure S3. — In vitro differentiation of aNSCs isolated from the subventricular zone of adult mice. Neurospheres that were expanded in neurosphere medium in the presence of growth factors were differentiated on PDL-coated coverslips in the presence of 5 % FCS in the absence of growth factors. On day 0, a very small proportion of cells in neurospheres were NPCs (DCX+), but none were OPCs (NG2+), maturing neuronal cells (NF200+), or oligodendrocytes (MBP+). The differentiation cultures were fixed after 2, 4, and 8 days and immunostained for indicated markers of neuronal, oligodendroglial, or astrocytic lineages. At day 2 of culture, differentiation to NPCs (DCX+), OPCs (NG2+), and astrocytes (GFAP+) were observed, which matured with time (day 4 and 8) to neuronal cells (β-tubulin III+), MBP-producing oligodendrocytes, or to astrocytes. Nuclei were visualized by DAPI counterstaining. Scale bar: 50 μm. (TIF 8316 kb) [file 12974_2015_468_MOESM3_ESM.tif]

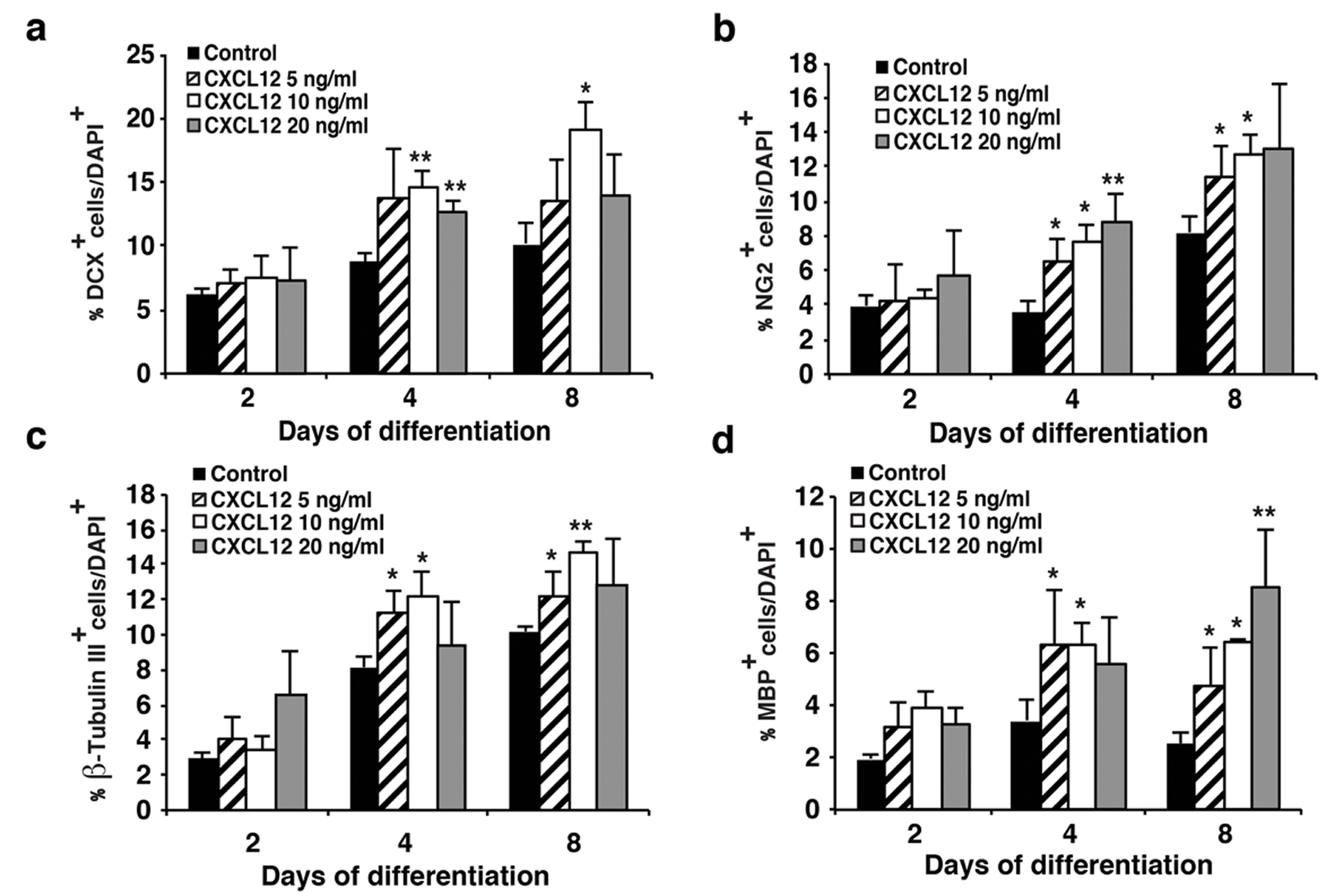

Supplement: Additional file 4: Figure S4. — CXCL12 promotes differentiation and maturation of aNSCs dose response. The data are from immunofluorescent staining for NPCs (DCX+, red) and OPCs (NG2+, green) (as in Fig. 7a) and for maturing neurons (βΙΙΙ-tubulin+, green) or oligodendrocytes (MBP+, red) (as in Fig.7d) of cultures of aNSCs undergoing differentiation in the absence or presence of CXCL12 (5, 10, or 20 ng/ml). The representative images for the effect of 10 ng/ml CXCL12, as an optimal dose, is shown in Fig. 7. The effects of the different concentration of CXCL12 on the in vitro differentiation and maturation of aNSCs are summarized here quantitatively. (a) Quantitative analysis of NPCs (DCX+). (b) Quantitative analysis of OPCs (NG2+). (c) Quantitative analysis of maturing neurons (βΙΙΙ-tubulin+). (d) Quantitative analysis of mature oligodendrocytes (MBP+). The effect of 5 ng/ml CXCL12 was lower than other concentrations; effects of 10 and 20 ng/ml CXCL12 were quite comparable, with 10 ng/ml being somewhat more optimal. We therefore showed in the body of the manuscript (Fig. 7) only the effects of CXCL12 at 10 ng/ml concentration. Results in a–d are presented as percentages of nuclei numbers and represent the mean ± SEM from three independent experiments. *p ≤ 0.05; **p < 0.01. (TIF 8921 kb) [file 12974_2015_468_MOESM4_ESM.tif]
